# Maternal high-cholesterol diet negatively programs offspring bone development and downregulates hedgehog signaling in osteoblasts

**DOI:** 10.1016/j.jbc.2022.102324

**Published:** 2022-08-02

**Authors:** SVVS Ravi Mangu, Kalpana Patel, Shinde Vijay Sukhdeo, M.R. Savitha, Kunal Sharan

**Affiliations:** 1 Department of Molecular Nutrition, CSIR-Central Food Technological Research Institute, Mysuru, India; 2 Academy of Scientific and Innovative Research (AcSIR), Ghaziabad, India; 3 Department of Meat and Marine Sciences, CSIR-Central Food Technological Research Institute, Mysuru, India; 4 Department of Paediatrics, Mysore Medical College and Research Institute, Mysuru, India

**Keywords:** maternal nutrition, fetal programming, hypercholesterolemia, osteoblast, osteoclast, hedgehog signaling, bone turnover markers, ALP, alkaline phosphatase, B.Ar, cortical cross-sectional area, BFR/BS, bone formation rate per bone surface, BMC, bone marrow cell, BMD, bone mineral density, B.Pm, cortical bone perimeter, BrdU, bromodeoxyuridine, BV/TV, bone volume/tissue volume, C, control, CB, cord blood, Col1a1, type I collagen A1, Cs.Th, cortical thickness, CTX, C-terminal type-1 collagen cross-links, E.Pm, endosteal perimeter, Gli, glioma-associated oncogene transcription factors, HC, high cholesterol, HDL, high-density lipoprotein, Ihh, Indian hedgehog, LDL, low-density lipoprotein, LV, lumbar vertebrae, MB, maternal blood, MCOB, mouse calvarial osteoblast, MMI, mean moment of inertia, N.Ob/Tb.B.Ar, number of osteoblasts per trabecular bone area, N.Oc/Tb.B.Ar, number of osteoclasts per trabecular bone area, N.Os/Tb.B.Ar, number of osteocytes per trabecular bone area, OCN, osteocalcin, Oc.S/BS, osteoclast surface per bone surface, OPG, osteoprotegerin, PINP, procollagen type 1-N terminal propeptide, RANKL, receptor activator of nuclear factor of kappa-B ligand, Runx2, Runt-related transcription factor 2, Shh, Sonic hedgehog, SMI, structural model Index, T.Pm, periosteal perimeter, Tb.B.Ar, trabecular bone area, Tb.N, trabecular number, Tb.Pf, trabecular pattern factor, Tb.sp, trabecular separation, Tb.Th, trabecular thickness, TRAP, tartrate-resistant acid phosphatase

## Abstract

Cholesterol is one of the essential intrauterine factors required for fetal growth and development. Maternal high cholesterol levels are known to be detrimental for offspring health. However, its long-term effect on offspring skeletal development remains to be elucidated. We performed our studies in two strains of mice (C57BL6/J and Swiss Albino) and human subjects (65 mother–female newborn dyads) to understand the regulation of offspring skeletal growth by maternal high cholesterol. We found that mice offspring from high-cholesterol-fed dams had low birth weight, smaller body length, and delayed skeletal ossification at the E18.5 embryonic stage. Moreover, we observed that the offspring did not recover from the reduced skeletal mass and exhibited a low bone mass phenotype throughout their life. We attributed this effect to reduced osteoblast cell activity with a concomitant increase in the osteoclast cell population. Our investigation of the molecular mechanism revealed that offspring from high-cholesterol-fed dams had a decrease in the expression of ligands and proteins involved in hedgehog signaling. Further, our cross-sectional study of human subjects showed a significant inverse correlation between maternal blood cholesterol levels and cord blood bone formation markers. Moreover, the bone formation markers were significantly lower in the female newborns of hypercholesterolemic mothers compared with mothers with normal cholesterolemic levels. Together, our results suggest that maternal high cholesterol levels deleteriously program offspring bone mass and bone quality and downregulate the hedgehog signaling pathway in their osteoblasts.

Cholesterol is an organic hydrophobic compound, and its biosynthesis takes place in every nucleated cell in the body (1). It maintains cell membranes' structural integrity and fluidity (2, 3). Cholesterol also acts as a precursor for bile salts and several hormones (4). While it is indispensable for various biological functions, elevated levels of total cholesterol in blood cause a pathological condition termed hypercholesterolemia (5, 6). Besides genetics, various lifestyle changes contribute to the risk of developing hypercholesterolemia, the most important being the high-cholesterol (HC) diet (7, 8, 9, 10). Complications associated with hypercholesterolemia include atherosclerotic cardiovascular diseases, obesity, increased resistance to insulin, hypertension, and osteoporosis (11, 12). Accumulating evidence from various research outputs shows an association between hypercholesterolemia and bone loss (13). Both animal and clinical studies have shown that high serum cholesterol is linked with reduced bone mineral density (BMD) and increased risk of fractures (14, 15, 16, 17, 18, 19, 20). The risk of decline in bone mass is more prominent in women than in men, particularly postmenopausal women (21, 22, 23).

Intrauterine environment and maternal nutrition are the crucial factors for fetal growth and development (24, 25). Any constraints during the critical period of fetal development may permanently alter the structure and function of the growing fetus (26). Cholesterol is a major nutritional factor that actively participates in fetal development through indigenous synthesis and maternal transfer (27, 28). It has been suggested that almost 20% of fetal cholesterol in the embryo is derived from maternal placental cholesterol pools, and this percentage is further higher in hypercholesterolemic mothers (29). Given that cholesterol is a source of hormones and its levels influence the placental supply of nutrients, hypercholesterolemia is detrimental to fetal development and growth (30). Importantly, maternal hypercholesterolemia predisposes individuals to a spectrum of metabolic and degenerative diseases at later stages of life through fetal programming (31, 32).

Even though cholesterol is indispensable for fetal development and hypercholesterolemia is a potential risk factor for osteoporosis, no study has evaluated the effect of maternal HC diet on the development of the offspring's skeleton. Moreover, there is a lack of understanding of the cellular and molecular mechanisms involved. Therefore, we investigated how maternal HC diet may interfere with bone formation from embryogenesis till adulthood of the offspring in a mice model. Furthermore, to validate our animal study's findings, we conducted a human cross-sectional study and correlated the maternal levels of cholesterol with offspring's cord blood bone turnover markers in full-term pregnancies.

## Results

### Effect of HC diet on maternal weight gain, lipid profile, litter size, and offspring's skeletal development at E18.5 days in C57BL6/J mice

**Figure 1:**
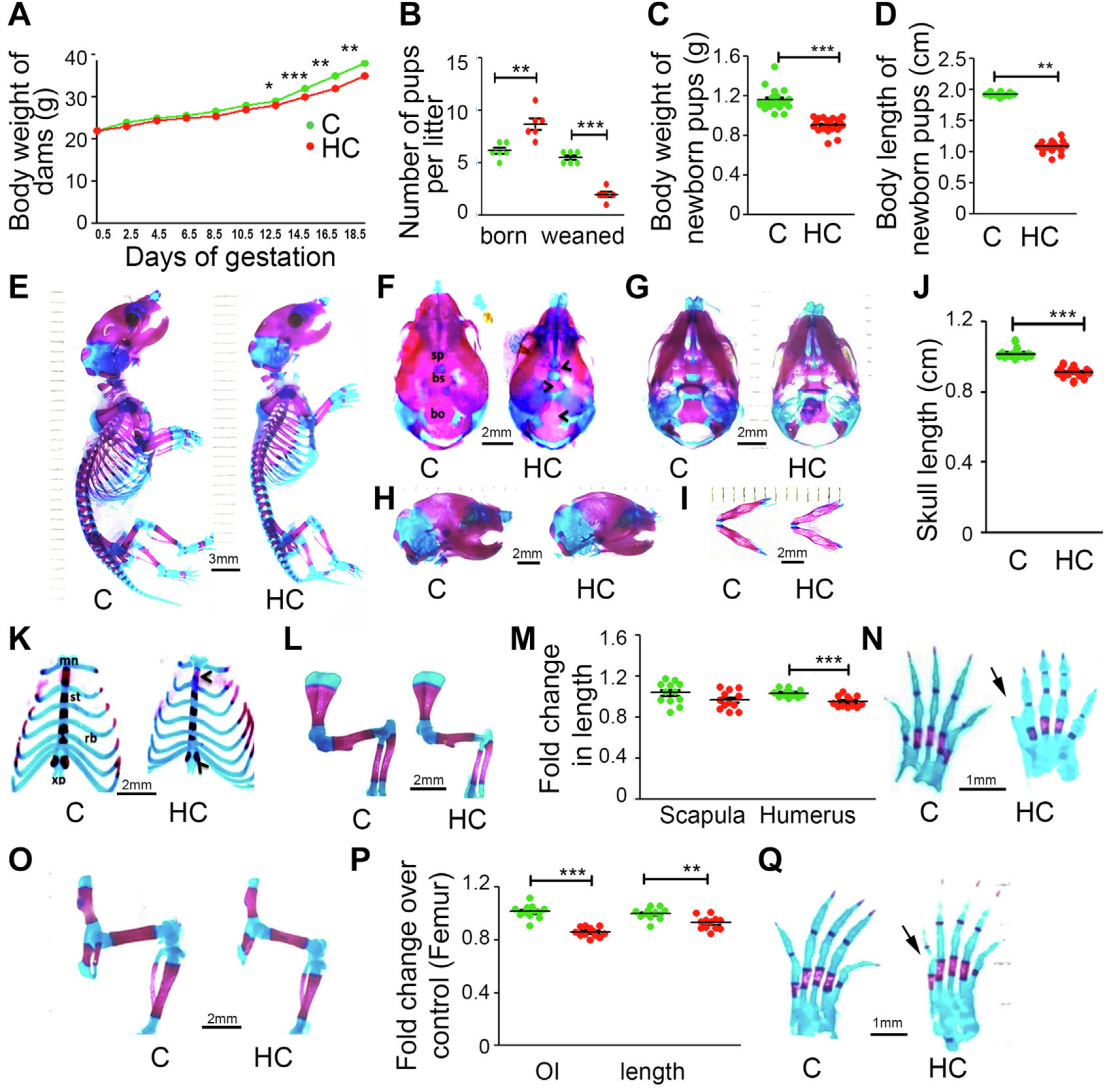
**Maternal HC diet causes poor embryonic skeletal development in C57BL6/J mice**. *A*, effect of C and HC diet on maternal gestational weight gain and (*B*) litter size. *C*, effect of maternal C and HC diet on newborn offspring’s body weight and (*D*) body length at birth. Representative *alizarin red* and *alcian blue*–stained images of (*E*) whole mount and skull ((*F*) dorsal side, (*G*) ventral side, (*H*) lateral side, and (*I*) mandible) of C and HC group embryo at E 18.5 days. *J*, fold change in skull length over C. Representative *alizarin red* and *alcian blue*–stained (*K*) sternum and (*L*) fore limb images. Fold change in length of (*M*) scapula and humerus over C. Representative *alizarin red* and *alcian blue*–stained (*N*) hind limb images. Fold change in (*O*) osteoid index and femur length over C. Representative *alizarin red* and *alcian blue*–stained images of (*P*) forepaw and (*Q*) hind paw. Scale bar as indicated in the images. Each group contains ≥6 animals. For *alcian blue*/*alizarin red* staining studies four litters with a minimum of three animals per litter were analyzed. Data are represented as mean±SEM (∗*p* < 0.05, ∗∗*p* < 0.01, ∗∗∗*p* < 0.001). C, control; HC, high cholesterol.

Female C57BL6/J mice were plugged and maintained on either Control (C) or HC diet throughout pregnancy and lactation. No difference in dietary consumption was found between the dams of C and HC groups (data not shown). However, there was a reduction in weight gain in the HC dams as compared with C dams during gestation (Fig. 1*A*). Moreover, feeding the HC diet to the plugged mice increased their serum and liver total cholesterol, triglycerides, and HDL- and LDL-cholesterol both at birth and weaning of their pups (Table S3). An increase in litter size was found in the HC group compared with C, but the number of pups weaned in the HC group was significantly lower than in C due to their poor survival after birth (Fig. 1*B*). A decrease in body weight and crown to rump length of the HC group newborn pups was found (Fig. 1, *C* and *D*). E18.5-day embryos were stained with alizarin red and alcian blue. As a cationic dye, alcian blue stains cartilage and appears in blue, whereas, as an anionic dye, alizarin red binds to calcium in bone and stains it red. After staining, they showed a gross reduction in the total length and mineralized bone in the HC group (Fig. 1*E*). Analysis of the skull, a site of intramembranous ossification, showed a reduction in the HC offspring's skull size compared with C (Fig. 1, *F*–*J*). Moreover, HC group offspring’s skull also had decreased mineralization in the dorsal (Fig. 1*F*), ventral (Fig. 1*G*), and lateral regions (Fig. 1*H*) with premature cranial sutures and smaller mandible (Fig. 1*I*) when compared with C group offspring. Analysis of the endochondral ossification sites showed a reduced calcification in the manubrium region and the xiphoid process of the sternum in offspring of HC-fed dams over control (Fig. 1*K*). Besides, the length and osteoid index of humerus and femur were significantly decreased in HC offspring compared with C (Fig. 1, *L*–*O*). Interestingly, a review of the pattern of digit formation from both forelimb and hind limb evidenced that they were incompletely derived and differentiated in the HC group’s offspring compared with C (Fig. 1, *P* and *Q*).

### Effect of maternal HC diet on cortical and trabecular bone microarchitecture in C57BL6/J offspring at 12 weeks of age

**Figure 2:**
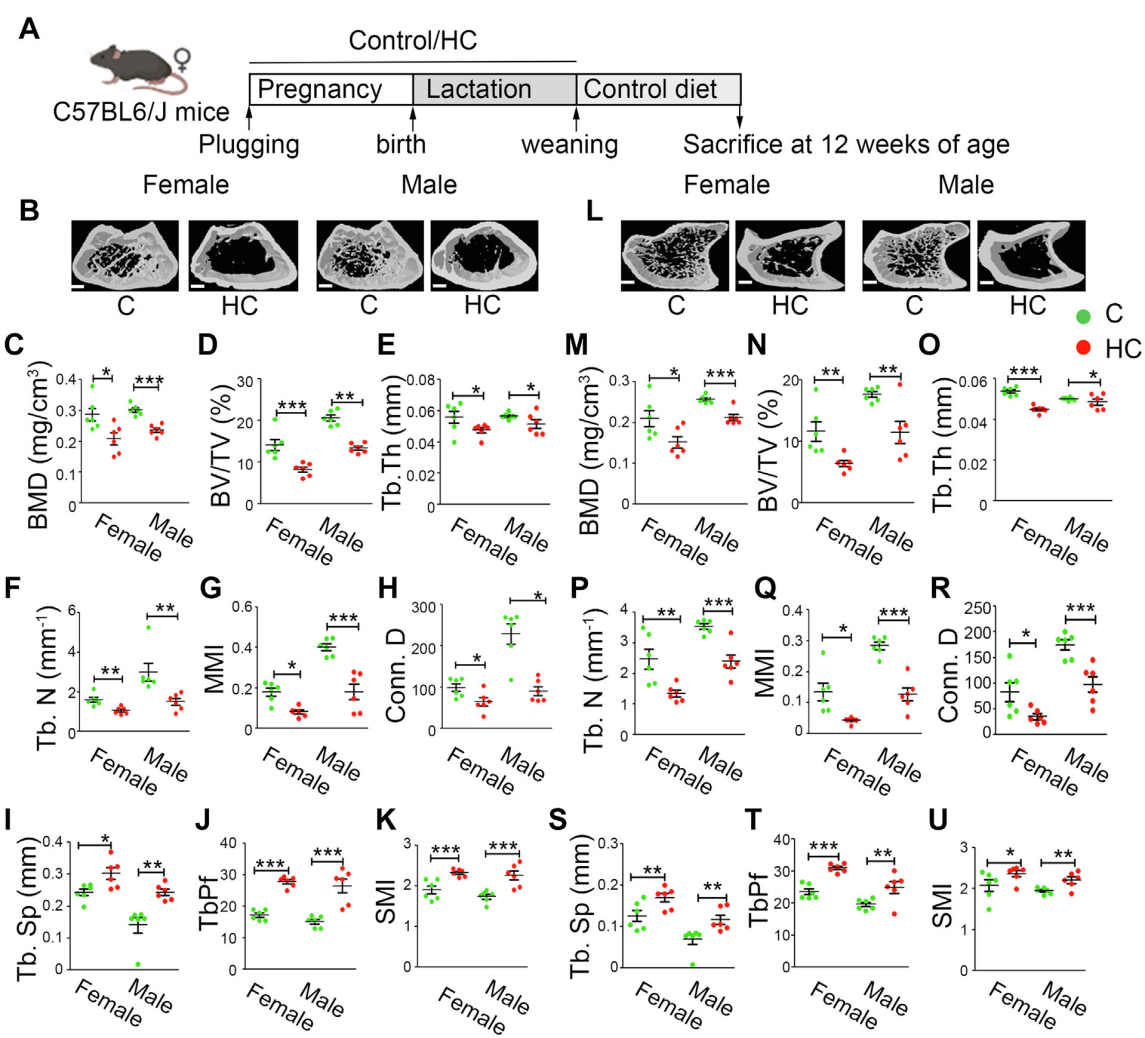
**Maternal HC diet results in impaired bone mass acquisition in the offspring of C57BL6/J mice**. *A*, schematic representation of experimental plan. *B*, representative reconstructed micro-computed tomography images of distal femur, (*C*) BMD (mg/cm^3^), (*D*) BV/TV (%), (*E*) Tb.Th (mm), (*F*) Tb.N (mm^-1^), (*G*) MMI, (*H*) Conn.D, (*I*) Tb.Sp (mm), (*J*) Tb.Pf, and (*K*) SMI of both C and HC offspring at 12 weeks age. (*L*) Representative reconstructed micro-computed tomography image of proximal tibia (*M*) BMD (mg/cm^3^), (*N*) BV/TV (%), (*O*) Tb.Th (mm), (*P*) Tb.N (mm^-1^), (*Q*) MMI, (*R*) Conn.Dn, (*S*) Tb.Sp (mm), (*T*) Tb.Pf, and (*U*) SMI of both C and HC offspring at 12 weeks age. The scale bar represents 250 μm for *B* and *L*. Each group contains ≥6 animals. Data are represented as mean ± SEM (∗*p* < 0.05, ∗∗*p* < 0.01, ∗∗∗*p* < 0.001). BMD, bone mineral density; BV/TV, bone volume/tissue volume; C, control; Conn.D, connection density; HC, high cholesterol; MMI, mean moment of inertia; SMI, structural model index; Tb.N, trabecular number; Tb.Pf, trabecular pattern factor; Tb.Sp, trabecular separation; Tb.Th, trabecular thickness.

We next investigated whether the HC group offspring recover from the *in utero* decrease in skeletal mineralization and development when grown on a C diet (Fig. 2*A*). As there was high mortality in the offspring of the HC group, we could only analyze their bones at 12 weeks of age. An increase in body weight and a decrease in body length was observed in both female and male HC group offspring compared with C (Table S4). The trabecular microarchitecture of the femur distal metaphysis and the tibia epiphysis was analyzed using reconstructed 3D micro-computed tomography (μ-CT) data. Femoral data of both gender offspring from HC showed significant deterioration in their bone microarchitecture (Fig. 2*B*). Furthermore, femur trabecular bone parameters, such as BMD, bone volume/tissue volume (BV/TV), trabecular thickness (Tb.Th), trabecular number (Tb.N), mean moment of inertia (MMI), and connection density (Conn.D), which are directly proportional to bone quality, were significantly decreased in the HC group (Fig. 2, *C*–*H*), whereas trabecular parameters, such as trabecular separation (Tb.Sp), trabecular pattern factor (Tb.Pf), and structure model index (SMI), which are inversely related to bone quality, were significantly increased in the offspring of the HC group compared with control (Fig. 2,*I*–*K*). Similarly, data obtained from the cortical bone at femur diaphysis revealed a significant decrease in the positive indicators of bone quality, *i.e.*, BMD, T.Ar (periosteal area), T.Pm (periosteal perimeter), B.Ar (cortical mean cross-sectional area), B.Pm (cortical bone perimeter), Cs.Th (cortical thickness), and MMI, whereas porosity, a negative indicator of bone quality, was significantly increased in both the genders of HC offspring (Table S4).

Analysis of tibia epiphysis also revealed a gross decrease in its trabecular microarchitecture (Fig. 2*L*), followed by a decrease in BMD, BV/TV, Tb.Th, Tb.N, MMI, and Conn.D (Fig. 2, *M*–*R*) and an increase in Tb.sp, Tb.Pf, and SMI (Fig. 2, *S*–*U*) in both genders of HC offspring. Cortical data at tibia diaphysis also showed a marked significant decrease in BMD, T.Ar, T.Pm, B.Ar, B.Pm, Cs.Th, and MMI with an increase in its porosity in offspring of the HC group of both gender compared with control. (Table S4).

### Effect of HC diet on maternal weight gain, lipid profile, litter size, and offspring's skeletal development at E18.5 days in Swiss Albino mice

**Figure 3:**
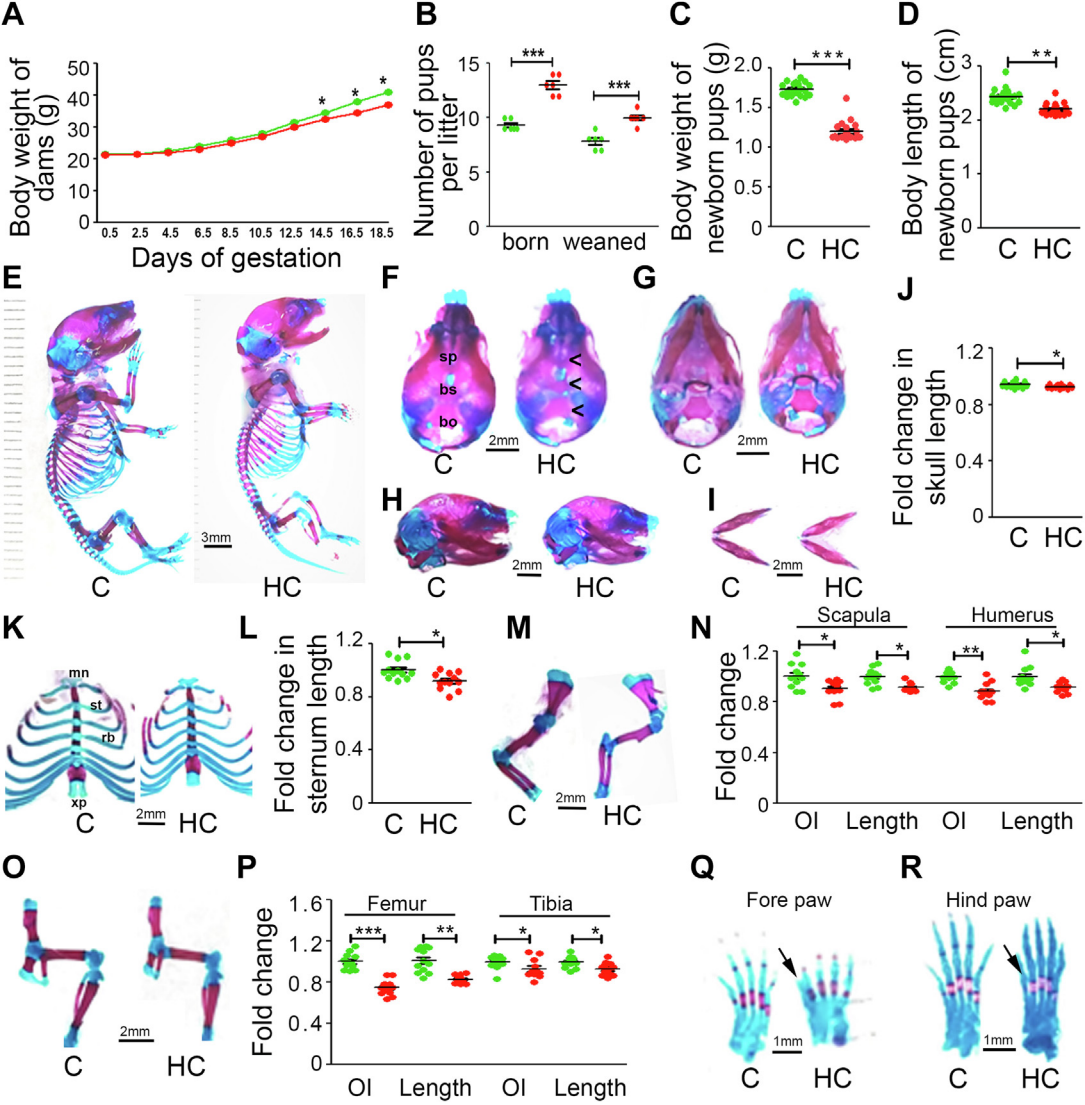
**Maternal HC diet causes reduced embryonic skeletal development in Swiss Albino mice**. *A*, effect of C and HC diet on maternal gestational weight gain and (*B*) litter size. (*C*) effect of maternal C and HC diet on newborn offspring’s body weight and (*D*) body length at birth. Representative *alizarin red* and *alcian blue*–stained images of (*E*) whole mount and skull ((*F*) dorsal side, (*G*) ventral side, (*H*) lateral side, and (*I*) mandible) of C and HC group embryo at E 18.5 days. (*J*) fold change in skull length over C. Representative *alizarin red* and *alcian blue*–stained images of (*K*) sternum and fold change in (*L*) length of sternum over C. (*M*) representative *alizarin red* and *alcian blue*–stained images of the forelimb. Fold change in (*N*) osteoid index and length of scapula and humerus over C. (*O*) representative *alizarin red* and *alcian blue*–stained images of Hind limb. Fold change in (*P*) osteoid index and length of femur and tibia over control. Representative *alizarin red* and *alcian blue*–stained images of (*Q*) forepaw and (*R*) hind paw. Scale bar as indicated in the images. Each group contains ≥6 animals. Data are represented as mean ± SEM (∗*p* < 0.05, ∗∗*p* < 0.01, ∗∗∗*p* < 0.001). C, control; HC, high cholesterol.

As there was a high mortality rate in maternal HC-fed C57BL6/J mice pups, we shifted our experiments to Swiss Albino mice, known for better maternal care. Like C57BL6/J mice, Swiss mice dams had no change in the dietary consumption between the two groups during pregnancy, and HC group dams gained less weight (Fig. 3*A*). Similarly, feeding HC diet to the plugged Swiss Albino mice also increased their serum and liver total cholesterol, triglycerides, and HDL- and LDL-cholesterol at birth and weaning of their pups (Table S3). A significant increase in litter size was observed in HC group compared with C. In addition, Swiss Albino mice pups from the HC group showed better survivability than C57BL6/J mice (Fig. 3*B*). Similar to C57BL6/J, both birth weight and body length were significantly reduced in the newborn offspring from HC-fed dams (Fig. 3, *C* and *D*). Skeletal preparation from E18.5-day embryos also showed similar results as C57BL6/J mice, where overall mineralization and length of the skull, sternum, and long bones of HC offspring were decreased compared with C offspring (Fig. 3, *E*–*P*). Interestingly, here also, an incomplete digital patterning of both forelimb and hind limb in the offspring of HC group was observed (Fig. 3, *Q* and *R*).

### Effect of maternal HC diet on the bone microarchitecture of the Swiss Albino mice offspring at later ages

**Figure 4:**
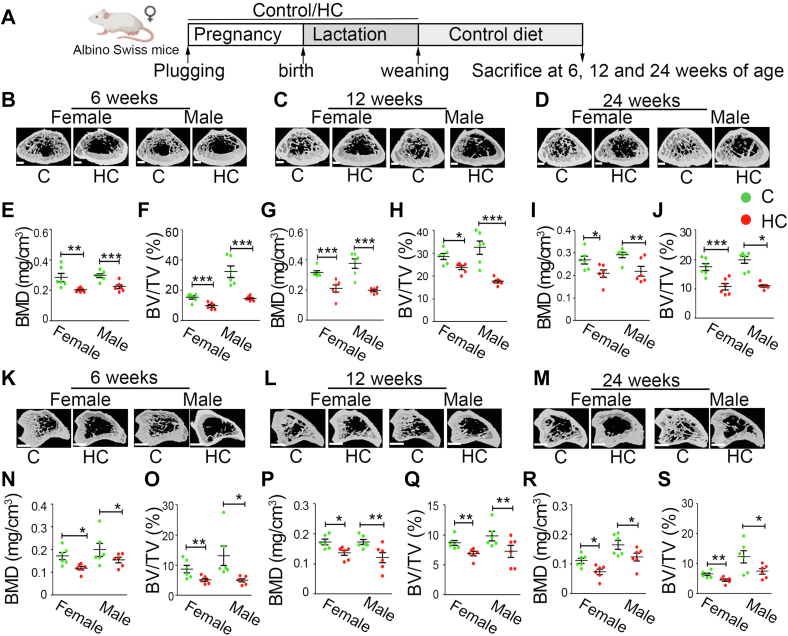
**Maternal HC diet deteriorates bone mass and causes growth retardation in Swiss Albino mice offspring**. *A*, schematic representation of the experimental plan. Representative micro-computed tomography images of the distal femur of offspring at (*B*) 6, (*C*) 12, and (*D*) 24 weeks of age. Femur BMD (mg/cm^3^) and BV/TV (%) of (*E* and *F*) 6-week, (*G* and *H*) 12-week, and (*I* and *J*) 24-week-old offspring. Representative micro-computed tomography image of the proximal tibia of offspring at (*K*) 6, (*L*) 12, and (*M*) 24 weeks of age. Femur BMD (mg/cm^3^) and BV/TV (%) of (*N* and *O*) 6-week, (*P* and *Q*) 12-week, and (*R* and *S*) 24-week-old offspring. The scale bar represents 250 μm for *B*–*D* and 450 μm for *K*–*M*. Each group contains ≥6 animals. Data are represented as mean ± SEM (∗*p* < 0.05, ∗∗*p* < 0.01, ∗∗∗*p* < 0.001). BMD, bone mineral density; BV/TV, bone volume/tissue volume; C, control; HC, high cholesterol.

Swiss Albino mice pups from both the groups exposed to either C or HC during pregnancy and lactation were weaned on a C diet and sacrificed at different stages of life, *i.e.*, young (6 weeks), adult (12 weeks), and late adult age (24 weeks) (Fig. 4*A*). There was an increase in the body weight and a decrease in the body length of the HC group offspring in all the age groups and both the genders (Table S5). 3D μ-CT data from the femoral trabecular region revealed that compared with C, a significant bone microarchitectural deterioration was observed in HC offspring, independent of their age and gender (Fig. 4, *B*–*D*). Femur trabecular BMD, BV/TV (Fig. 4, *E*–*J*), Tb.Th, Tb.N, MMI, and Conn.D (Table S5) were significantly decreased, whereas Tb.Sp, Tb.Pf, and SMI were significantly increased in the offspring of HC group. Analysis of cortical bone at femur diaphysis revealed a significant decrease in the BMD, T.Ar, T.Pm, B.Ar, B.Pm, Cs.Th, and MMI, whereas porosity was significantly increased in both the genders of HC offspring (Table S6).

Similar to the femur, tibia trabeculae of the HC group offspring also showed significant deterioration in their bone microarchitecture due to reduced BMD, BV/TV, Tb.Th, Tb.N, MMI, and Conn.Dn with an increase in Tb.Sp, Tb.Pf, and SMI in the HC group offspring compared with C (Fig. 4, *K*–*S*). Data obtained from analysis of cortical bone at femur and tibia diaphysis also revealed a consistent and significant decrease in BMD, T.Ar, T.Pm, B.Ar, B.Pm, Cs.Th, and MMI with a significantly increased porosity in both the genders of HC offspring compared with control (Table S6).

### Effect of maternal HC diet on the vertebral histology and bone remodeling in the Swiss Albino mice offspring

**Figure 5:**
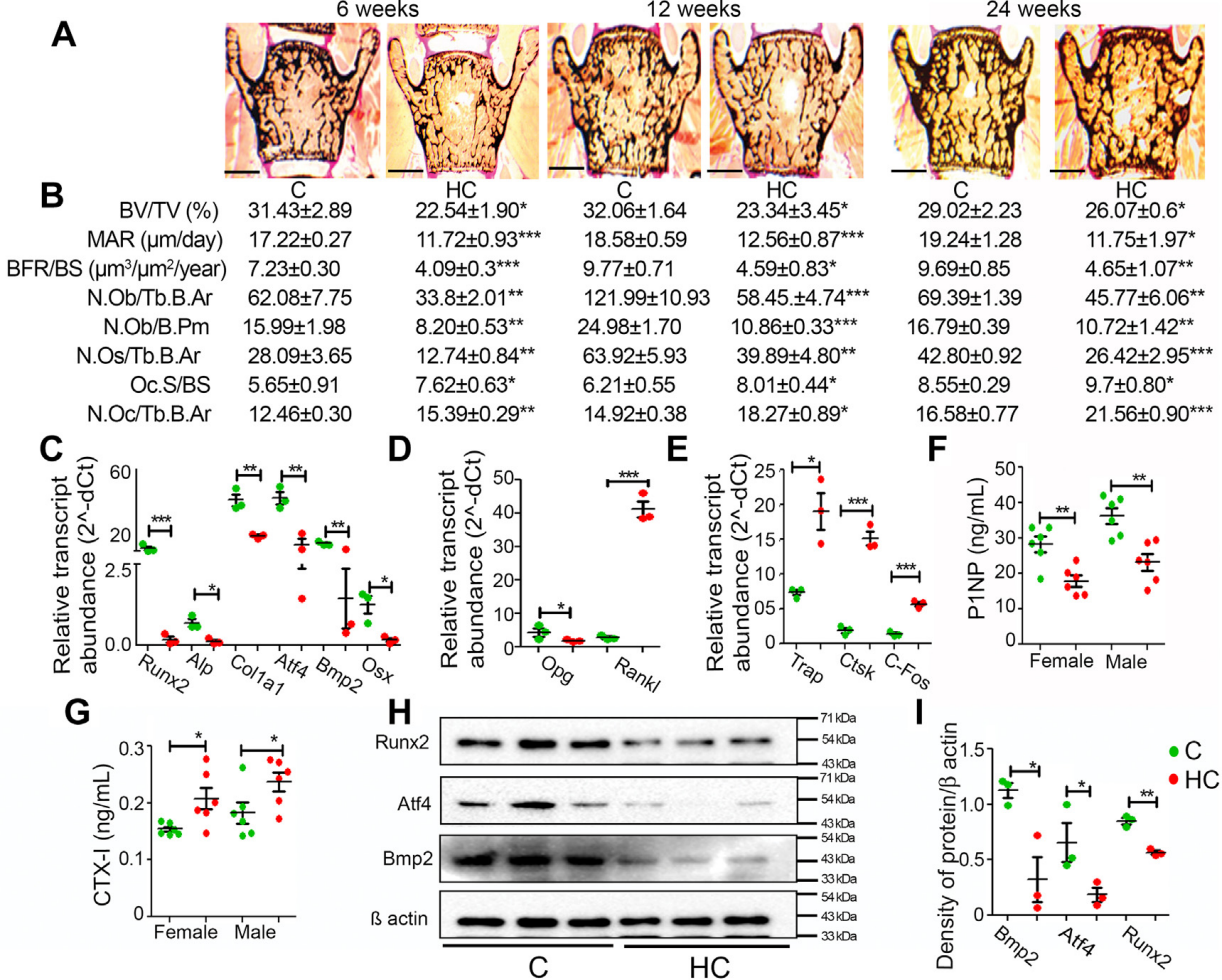
**Maternal HC diet decreases bone formation and increases bone resorption in Swiss Albino mice offspring**. *A*, representative images of von Kossa-stained sections of female offspring’s LV4 at 6, 12, and 24 weeks of age (the scale bar represents 500 μm). *B*, histomorphometric analysis of BV/TV, MAR, BFR/BS, N.Ob/Tb.B.Ar, N.Ob/B.Pm, N.Os/Tb.B.Ar, Oc.S/BS, and N.Oc/Tb.B.Ar. *C*, relative transcript abundance in mRNA expression of osteoblast marker genes *Runx2*, *Alp*, *Col1a1*, *Atf4*, *Bmp2*, *and Osx*, and (*D*) relative transcript abundance in mRNA expression of *Opg and Rankl* in the long bones of 24-week-old female offspring. *E*, relative transcript abundance in mRNA expression of osteoclast marker genes *Trap*, *Ctsk*, and *C-fos* mRNA expression. *F*, serum P1NP and (*G*) urinary CTX-1 levels in 24-week-old female and male offspring. *H*, Western blot analysis of Bmp2, Atf4, and Runx2 and (*I*) densitometric measurements from the long bones of 24-week-old offspring. Each group contains ≥6 animals. Data are represented as mean ± SEM (∗*p* < 0.05, ∗∗*p* < 0.01, ∗∗∗*p* < 0.001). BFR/BS, bone formation rate per bone surface; BV/TV, bone volume/tissue volume; C, control; HC, high cholesterol; MAR, mineral apposition rate; N.Ob/B.Pm, osteoblast number per bone perimeter; N.Ob/Tb.B.Ar, number of osteoblasts per trabecular bone area; N.Oc/Tb.B.Ar, number of osteoclasts per trabecular bone area; N.Os/Tb.B.Ar, number of osteocytes per trabecular bone area; Oc.S/BS, osteoclast surface per bone surface.

Histological analysis was performed on lumbar vertebra 4 (LV4) of female offspring of 6, 12, and 24 weeks of age. Von Kossa staining of sections revealed a significant decrease in HC offspring’s BV/TV (Fig. 5*A*). Calcein double-labeling analysis showed a reduction in mineral apposition rate and bone formation rate per bone surface (BFR/BS) in HC group vertebra. Further analysis revealed a significant decline in osteoblast number per trabecular bone area (N.Ob/Tb.B.Ar), osteoblast number per bone perimeter (N.Ob/B.Pm), and osteocyte number per trabecular bone area (N.Os/Tb.B.Ar), whereas a significant increase was observed in osteoclast surface/bone surface (Oc.S/BS) and osteoclast number per trabecular bone area (N.Oc/Tb.B.Ar) in HC group vertebra (Fig. 5*B*).

Next, a quantitative PCR (qPCR) analysis was performed with the RNA isolated from the long bones of 24-week female offspring. Key regulatory markers involved in bone formation and resorption were investigated. We found a significant decrease in the expression of osteogenic genes *Runx2, Alp, Col1a1, Atf4, Bmp2, and Osx* in the HC group (Fig. 5*C*). *Opg*, which protects bone from excessive resorption, was downregulated, and *Rankl*, which regulates and activates osteoclast formation, was significantly upregulated in the HC group (Fig. 5*D*). Furthermore, markers specific to osteoclasts and bone resorption were significantly upregulated in the HC group bones compared with the control (Fig. 5*E*). Alongside, serum Pro-Collagen Type 1 N terminal propeptide (P1NP, a bone formation marker) levels were significantly reduced (Fig. 5*F*), whereas C-Terminal type-1 collagen cross-links (CTX), a bone resorption marker, were significantly increased in HC group offspring (Fig. 5*G*).

Similar to mRNA, protein expression analysis from long bones of 24-week female offspring showed a significant decrease in the expression of Runx2, Atf4, and Bmp2 (Fig. 5,*H* and *I*) in the HC group compared with C.

### Effect of maternal HC diet on *ex vivo* cultured offspring's bone cells

**Figure 6:**
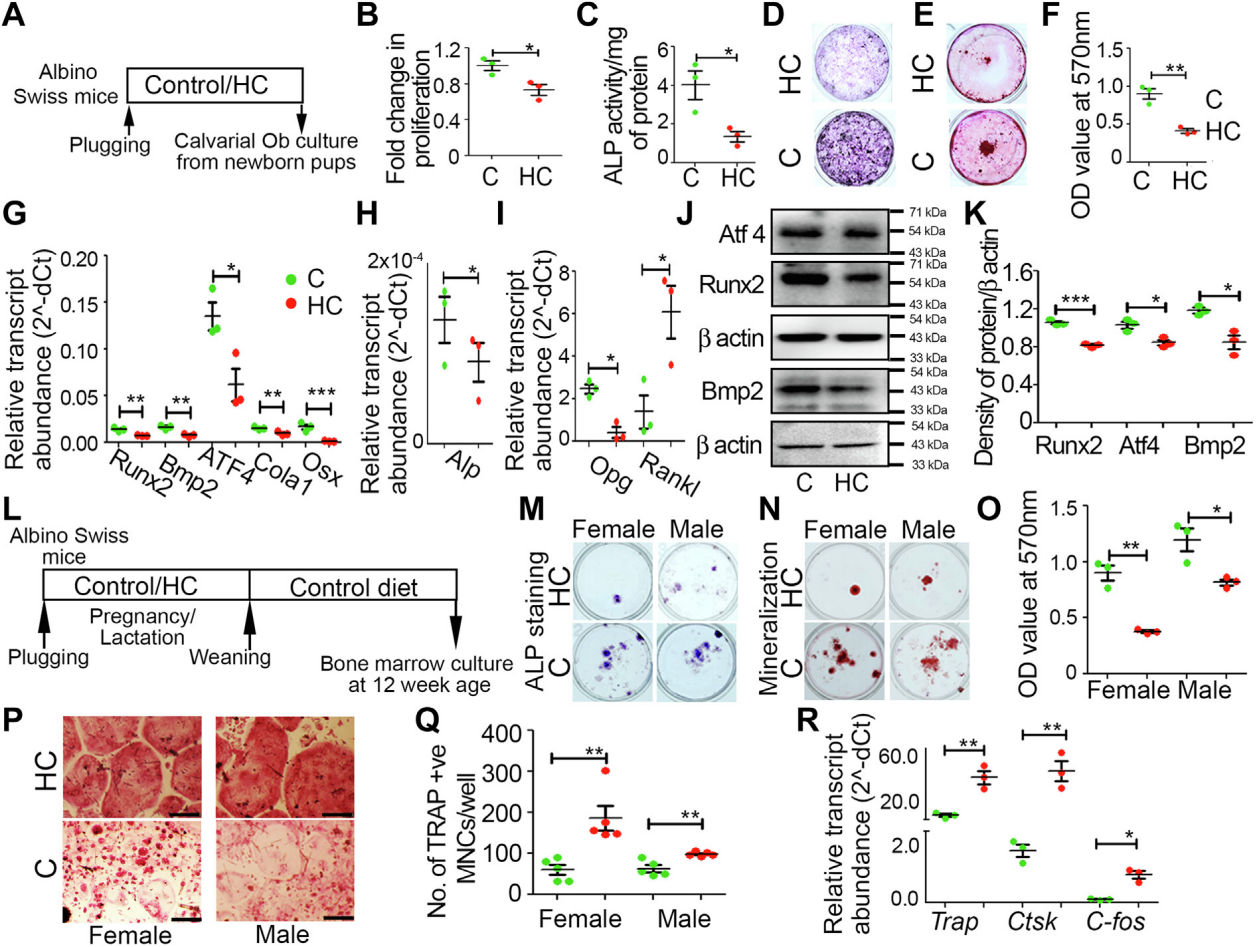
**Maternal HC diet decreases osteoblast cell function and enhances osteoclast differentiation in the offspring**. *A*, schematic representation of experimental plan for *ex vivo* MCOBs culture. *B*, fold change in proliferation by BrdU incorporation assay. *C*, ALP colorimetric assay expressed as ALP activity/mg protein. *D*, ALP staining. *E*, alizarin red staining of mineralized calcium nodules and (*F*) its quantification by cetyl pyridinium chloride extraction method. *G*, relative transcript abundance in mRNA expression of *Runx2*, *Col1a1*, *Bmp2*, *Atf4*, *and Osx*. *H*, relative transcript abundance in mRNA expression of *Alp. I*, relative transcript abundance in mRNA expression of *Opg and Rankl* in *ex vivo* cultured MCOBs. *J*, Western blot analysis and (*K*) densitometric measurements of Bmp2, Atf4, and Runx2 expression in *ex vivo* cultured MCOBs. *L*, schematic representation of experimental plan for *ex vivo* bone marrow cell culture. *M*, ALP staining, (*N*) *alizarin red* staining of mineralized calcium nodules, and (*O*) quantification by cetyl pyridinium chloride extraction of BMCs differentiated toward osteoblast. *P*, representative images of TRAP+ve multinucleated osteoclast cells of *ex vivo* cultured BMCs differentiated toward osteoclast (the scale bar represents 100 μm). *Q*, number of multinucleated osteoclast cells/well, performed in 96-well plates. *R*, relative transcript abundance in mRNA expression of osteoclast specific genes of *ex vivo* cultured BMCs differentiated toward osteoclast. Each experiment was repeated three times with a minimum of three replicates each. Data are represented as mean ± SEM (∗*p* < 0.05, ∗∗*p* < 0.01, ∗∗∗*p* < 0.001). ALP, alkaline phosphatase; BMC, bone marrow cell; C, control; HC, high cholesterol; MCOB, mouse calvarial osteoblast.

Primary mouse calvarial osteoblasts (MCOBs) were cultured from newborn pups of both C and HC-fed dams (Fig. 6*A*) to evaluate the cellular and molecular changes that take place during birth, responsible for their bone phenotype. We found a significant decrease in cell proliferation, differentiation, and mineralization in the MCOBs of the HC group compared with C (Fig. 6, *B*–*F*). Further qPCR with the RNA isolated from MCOBs displayed a significant decrease in osteoblast-specific markers *Runx2, Alp, Col1a1, Bmp2, Atf4, and Osx* (Fig. 6, *G* and *H*). Here also, *Opg* was significantly reduced, and *Rankl* expression was significantly higher in the HC group (Fig. 6*I*). In addition, Western blot analysis revealed a significant decrease in the expression of Bmp2, Atf4, and Runx2 in HC group MCOBs compared with C (Fig. 6, *J* and *K*).

Furthermore, we performed *ex vivo* bone marrow cell (BMC) culture from C and HC offspring at 12 weeks of age and evaluated their potential to differentiate toward osteoblast and osteoclast (Fig. 6*L*). A significant decrease in differentiation (Fig. 6*M*) and mineralization was found in both the genders of HC group offspring’s BMCs differentiated toward osteoblasts (Fig. 6, *N* and *O*). Results from the *ex vivo* BMCs differentiated toward osteoclast showed a significant increase in the tartrate-resistant acid phosphatase (TRAP)-positive multinucleated osteoclasts population in both genders of HC group offspring compared with C (Fig. 6, *P* and *Q*). qPCR analysis of osteoclast marker genes from the *ex vivo* cultured osteoclast cells showed a significant increase in the expression of *Trap*, *Ctsk*, and *c-fos* (Fig. 6*R*) in the HC group cells compared with C.

### Effect of maternal HC diet on hedgehog signaling in the *ex vivo* cultured MCOBs and long bones of the offspring

**Figure 7:**
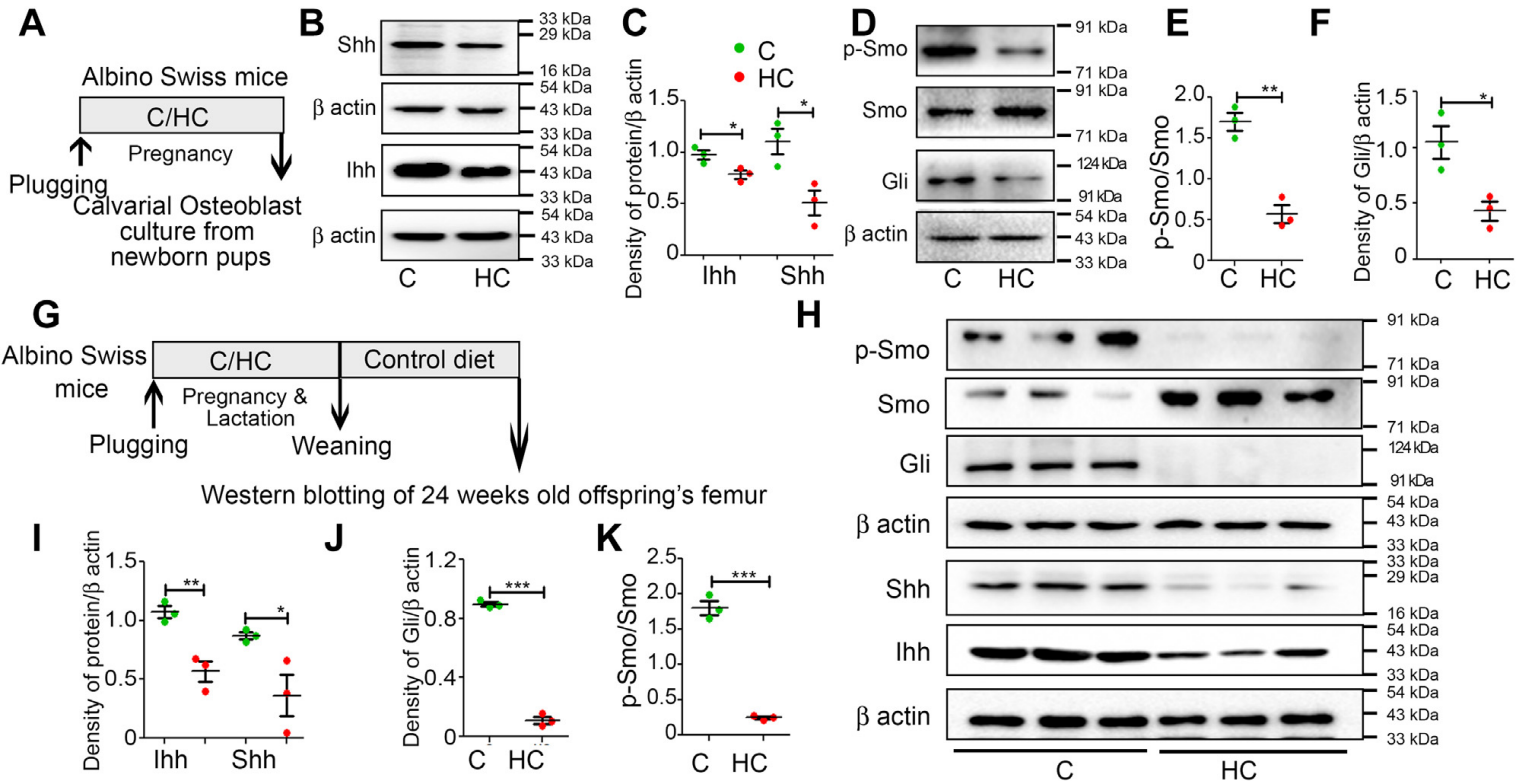
**Maternal HC diet negatively regulates hedgehog signaling in offspring’s bones.***A*, schematic representation of experimental plan for *ex vivo* mouse calvarial osteoblast culture studies. Western blot analysis of (*B*) Ihh and Shh from *ex vivo* cultured mouse calvarial osteoblasts and their (*C*) densitometric measurements. Western blot analysis of (*D*) Gli, Smo, and p-Smo. Densitometric analysis of (*E*) p-Smo/Smo ratio and (*F*) Gli. (*G*) schematic representation of the experimental design for protein expression from long bones of 24-week-old female offspring. *H*, Western blot analysis of Ihh, Shh, Gli, Smo, and p- Smo. *I*, densitometric analysis of Ihh, Shh, (*J*) Gli, and (*K*) p-Smo/Smo ratio. The β-actin (*lower*) for 7H is same β-actin shown in Figure 5*H* because Ihh and Shh proteins were blotted from the same gel. Each experiment was repeated 3 times with a minimum of three replicates each. Data are represented as mean ± SEM (∗*p* < 0.05, ∗∗*p* < 0.01, ∗∗∗*p* < 0.001). C, control; HC, high cholesterol.

As cholesterol acts as a posttranslational adduct of ligands involved in hedgehog signaling, which are known to affect skeletal development and maintenance, we next determined the influence of maternal HC on hedgehog signaling in the offspring’s bones. Western blotting was performed from the MCOB’s protein harvested from C and HC groups (Fig. 7*A*). We found a significant decrease in the expression of both ligands, Indian hedgehog (Ihh) and sonic hedgehog (Shh) (Fig. 7, *B* and *C*), in the HC group MCOBs. Moreover, the downstream signaling was also inhibited, evidenced by a reduction in Smo phosphorylation and a decline in glioma-associated oncogene transcription factor (Gli) levels (Fig. 7, *D*–*F*).

Furthermore, we assessed the expression levels of hedgehog proteins from long bones of 24-week female offspring (Fig. 7*G*). Similar to MCOBs results, a significant decrease in Ihh and Shh, followed by a decrease in Smo phosphorylation, and downregulation of Gli (Fig. 7, *H*–*K*) was observed in HC group bones compared with C.

### Effect of maternal hypercholesterolemia on biomarkers of bone formation and resorption in cord blood of female offspring at birth in human subjects

**Figure 8:**
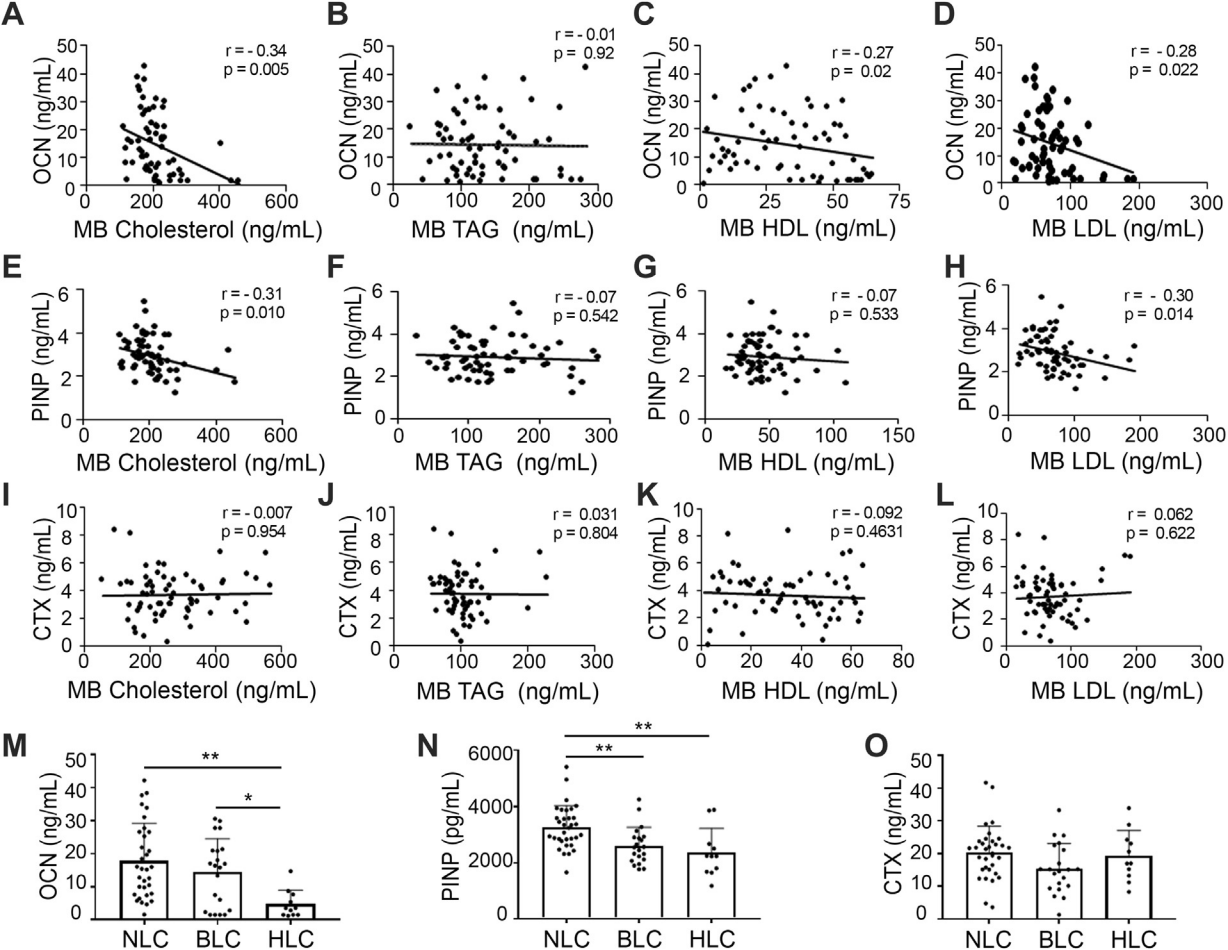
**Maternal hypercholesterolemia results in reduced levels of bone formation markers in the newborn offspring of human subjects**. Correlation studies between CB-OCN and MB- (*A*) total cholesterol, (*B*) TAG, (*C*) HDL-cholesterol, and (*D*) LDL-cholesterol. Correlation studies between CB-PINP and MB- (*E*) total cholesterol, (*F*) TAG, (*G*) HDL-cholesterol, and (*H*) LDL-cholesterol. Correlation studies between CB-CTX and MB- (*I*) total cholesterol, (*J*) TAG, (*K*) HDL-cholesterol, and (*L*) LDL-cholesterol. Pearson's r-value and “*p*” values are shown in the graph, the number of XY pairs (n) = 65. CB bone formation markers plot for (*M*) OCN and (*N*) P1NP and CB bone resorption marker (*O*) CTX plot from the newborns born to mothers with normal blood cholesterol (NLC), borderline blood cholesterol (BLC), and hypercholesterolemia (HLC). Significance between groups was determined by one-way ANOVA followed by Tukey's post hoc test. Data are represented as mean ± SD (∗*p* < 0.05, ∗∗*p* < 0.01). CB, cord blood; CTX, C-terminal type-1 collagen cross-links; HDL, high-density lipoprotein; LDL, low-density lipoprotein; MB, maternal blood; OCN, osteocalcin; PINP, procollagen type 1-N terminal propeptide; TAG, triacylglycerol.

The mice study showed that increased maternal cholesterol levels due to HC diet can modulate offspring’s bone mass. We collected MB-CB pairs from normal term deliveries to analyze whether similar regulation occurs in humans. Baseline clinical characteristics of the subjects are mentioned in Table S7. Maternal serum samples were subjected to lipid level analysis. Based on maternal cholesterol levels, samples were classified into three groups, i.e., normal cholesterol (160–200 mg/dl), borderline cholesterol (200–240 mg/dl), and high cholesterol >240 mg/dl. Furthermore, we analyzed bone turnover markers from the serum of cord blood samples. Correlation analysis of CB serum bone formation marker osteocalcin (OCN) showed a significant inverse correlation with MB serum total cholesterol (r = −0.34, *p* = 0.005), HDL-cholesterol (r = −0.27, *p* = 0.02), and LDL-cholesterol (r = −0.28, *p* = 0.028) levels, whereas no correlation was found with triacylglycerol (Fig. 8, *A*–*D*). Similar to OCN, P1NP (Fig. 8, *E* and *H*) also showed a significant inverse correlation with MB total cholesterol (r = −0.31, *p* = 0.014) and LDL-cholesterol (r = −0.30, *p* = 0.014). However, CB–procollagen type 1-N terminal propeptide (PINP) did not show any correlation with MB triacylglycerol and HDL-cholesterol (Fig. 8, *F* and *G*). Interestingly, correlation analysis with CB bone resorption marker, CTX with MB lipids, had no statistical significance (Fig. 8, *I*–*L*).

We next divided the MB-CB pairs into three groups: normal cholesterol, borderline cholesterol, and hypercholesterolemic, according to maternal cholesterol levels (Table S7). Analysis of CB-OCN, PINP, and CTX according to these groups showed a significant decline in bone formation markers (OCN and PINP) in borderline cholesterol and hypercholesteremic when compared with normal cholesterol, whereas no significant difference was found in CB-CTX levels (Fig. 8, *M*–*O*).

## Discussion

Maternal nutrition has marked implications on offspring's growth and development (33). Nutritional insults during pregnancy and lactation alter both prenatal and postnatal growth trajectories of offspring (34, 35). Research findings from epidemiological data and community-based cohort studies have shown (36, 37) long-term detrimental effects of high maternal cholesterol on offspring’s phenotype (37, 38). Here, in this study, we demonstrated how maternal HC diet regulates offspring bone homeostasis. We used two strains of mice, C57BL6/J and Swiss Albino, for the animal studies, as there was a low survival of C57BL6/J pups due to poor maternal care in the HC group. Moreover, we validated our results in human subjects through a cross-sectional study in MB-CB dyads.

Studies show that disproportionate or high dietary intake leads to improper placental ratios, which impair placental supply capacity and increase oxidative stress on a growing fetus. This results in reduced intrauterine growth rate, low birth weight, and less survivability (39, 40, 41, 42, 43). Results from both C57BL6/J and Swiss Albino mice showed a significant reduction in the weight gain during gestation in HC-fed dams compared with control. Moreover, in both the mice strains the HC group newborn’s body length and body weight were reduced. Lipid comparison between various species has shown that humans, hamsters, and mice have closer lipid fingerprints. Hamsters and mice have a closer cholesterol and triglycerides profile with humans than other species (44). A major difference exists in terms of circulating HDL and LDL levels and their metabolism. In humans, most of the cholesterol transport occurs in the form of LDL-cholesterol, whereas in mice, it is in the form of HDL-cholesterol. Another difference between mice and humans is that mice can obtain more cholesterol from their mothers during intrauterine growth, leading to the survival of even cholesterol-free mice for the first few generations (45, 46). Reports also suggest that, through dietary and genetic modifications, we can mimic the human pathophysiology in mice and a shift from HDL to LDL, primarily through a modified diet (46, 47). In our results also, we found that normal diet–fed dams had higher HDL-cholesterol levels than the LDL levels, but HC diet led to a pronounced increase in the LDL-cholesterol levels.

Interestingly, there was an increase in the litter size of HC dams. However, the pups in the HC group of C57BL6/J showed a very low survival after birth till weaning due to cannibalism of newborn pups. This might be because maternal high cholesterol levels are known to cause hypertension leading to poor maternal care and cannibalism (48). The pups' survival was better in the Swiss Albino mice as they have good maternal instincts. Further analysis of the offspring’s *in utero* skeletal development at E18.5 revealed intrauterine growth restriction and delayed ossification at both intramembranous and endochondral ossification sites in the HC group in both the strains of mice. Uterine crowding, a factor raised because of increased litter size and competition for nutrients with a depletion in placental nutrient shipment, might be a possible factor for reduced skeletal size in progeny (49). Moreover, maternal high cholesterol majorly exerts increased oxidative stress. It also causes changes in vascular reactivity that interrupts the supply of oxygen and nutrients to the fetus by placental transport (50, 51), which may lead to intrauterine growth restriction. We also observed an improper limb patterning and fusion of digits d4 and d5 in both fore and hind limbs of HC group offspring from both mice strains. This may be due to the HC levels that might have affected the limb bud formation and digit patterning during embryonic development by downregulating the genes and morphogens that are involved in limb bud and digit formation.

Adverse prenatal events may perturb postnatal growth rates (52). Furthermore, nutritional insults during the critical *in utero* development can permanently affect the bone mass (53, 54). Some reports also suggest that intrauterine growth restriction observed at the time of birth can reverse at later ages due to catch-up growth (55, 56, 57). We investigated what effect would occur in HC group offspring’s skeleton if switched to C diet at weaning. For that, the mice exposed to HC diet for gestation and lactation were transferred to the control diet at weaning and were maintained until they reached 6, 12, and 24 weeks of age. These age groups were selected to study the effect of maternal HC on offspring’s bone health at various stages of their life (young, adult, and late adult). We found that the HC group offspring of both the genders and at all ages, failed to recover even after switching to control diet at weaning. A reduction in both bone mass and bone quality, in both genders and at all the ages, was observed in maternal HC group offspring when compared with C. Even the linear growth of the HC group offspring was impaired. Together, these results suggested that the maternal HC results in a permanent and nonreversible skeletal impairment in the offspring.

Bone remodeling is a dynamic process involving both bone formation and resorption. Low bone mass disorders like osteoporosis can occur either by compromised bone formation, enhanced bone erosion, or both. To comprehend which of the two processes were affected in HC group offspring, histology of female offspring’s LV4 was performed. We found a reduction in BV/TV in the vertebra at all the age groups in the HC group offspring. The decreased bone mass resulted from the reduced bone formation (due to a decline in the osteoblast cells) and increased bone-resorbing osteoclast cells. qPCR analysis from the long bones also showed a decrease in osteoblast-specific markers with a concomitant increase in osteoclast-specific markers. Further analysis of serum bone turnover markers P1NP and CTX complemented these results. Data from the Western blotting also showed a significant decrease in the expression of genes essential for osteoblast maturation in HC group bones. These results were also complemented by the *ex vivo* cell culture experiments and provided evidence of a cell-autonomous effect. *Ex vivo* culture of MCOBs from newborn offspring showed a reduced osteoblast activity and associated marker genes expression. Moreover, the reduction in osteoprogenitor functions was seen even in the adult BMCs of HC group offspring. Osteoprotegerin (OPG) and receptor activator of nuclear factor of kappa-B ligand (RANKL) are factors produced by osteoblast and are important for osteoclast maturation. Higher levels of RANKL and low levels of OPG indicate osteoclast formation (58). mRNA levels showed reduced OPG and enhanced RANKL expression in HC group MCOs and bones compared with control. Consequently, *ex vivo* BMC experiments from the offspring showed increased osteoclastogenesis in the HC group. Together, these data provided evidence that offspring from the HC group failed to attain peak bone mass due to reduced osteoblast function and increased osteoclast activity.

Cholesterol is a crucial modulator of the hedgehog signaling pathway (59), vital for regulating various developmental events from embryonic stages (60). Reports suggest that its role is indispensable in bone metabolism from fetal stages till adulthood (61, 62). Ihh promotes osteogenesis in conjunction with bone morphogenic proteins (63, 64). During endochondral ossification, Ihh is essential for osteoblast differentiation from perichondral progenitor cells (65, 66). Shh, another morphogen, is elemental in patterning of limb buds, skeleton, and digit from the early stages of embryonic limb development (67). The progenitor cells that receive paracrine effects of Shh contribute to digit formation of d2, d3, d4, and d5 (64, 68). Gli is another critical factor for limb bud formation (69). Any mutations or improper regulation of hedgehog signaling leads to abnormal digit identity or truncation in limb bud formation (59, 64). We investigated the effect of maternal HC on morphogens and proteins of the hedgehog signaling pathway in *ex vivo* cultured MCOBs and adult long bones of the offspring. Our results found a decrease in the expression of both Ihh and Shh in MCOBs and adult mice bones of HC group offspring. The reduced expression of Ihh and Shh was accompanied by downregulation of downstream hedgehog signaling (Gli expression and Smo phosphorylation). The reduced expression of Bmp2 and Runx2 (the master regulator of osteoblast differentiation) in both *ex vivo* cultured MCOBs and adult long bones of HC group offspring can be explained by these results, as hedgehog signaling is a known modulator of their expression (70, 71, 72, 73). Although these results indicate a possible role of hedgehog signaling in the bone phenotype of the HC offspring, it is still unclear whether the downregulation of the signaling is causative or correlative to the decreased bone mass. Further research in this direction is required to better understand the mechanism of this phenomenon.

A systematic, integrated study of bone microarchitecture with bone turnover markers has great potential in the early assessment of bone development and fracture risk (74). Bone turnover markers are highly specific and act as surrogates to monitor the changes in bone mass during pregnancy (75). To validate our *in vivo* data in human subjects, we collected MB-CB pairs from normal, borderline cholesterol, and hypercholesteremic mothers at the time of delivery and compared their MB cholesterol levels with CB bone turnover markers. Our correlation analysis and linear regression assessment in healthy female newborns showed a significant inverse correlation between bone formation marker OCN and maternal total cholesterol, LDL-cholesterol, and HDL-cholesterol levels. Similarly, P1NP, another bone formation marker, also showed a significant inverse correlation with maternal cholesterol and LDL-cholesterol levels. At the same time, no statistically significant correlation was observed with bone resorption marker CTX. We also found a significantly lower level of bone formation markers in the offspring CB born to hypercholesterolemic mothers when compared with the CB of normal mother’s offspring. However, no significant difference was found in CTX between the groups. No change in CTX might be due to the fact that, at birth, there is more bone formation than bone resorption (76) and the difference may start to appear at later stages. Therefore, in the future, longitudinal studies following the offspring’s bone turnover markers or BMD at different life milestones would be required to shed more light on the phenomenon.

In conclusion, maternal exposure to HC diet during pregnancy in mice leads to intrauterine delay in skeletal growth. Maternal HC diet during pregnancy and lactation in mice also causes a permanent impairment of bone growth with no recovery at different stages of life, even when the offspring is switched to a regular diet. Maternal HC diet interferes with the mechanisms of bone remodeling by inhibiting bone formation and enhancing bone resorption. In MCOBs and adult mice bones, the effect seems to be through the inhibition of hedgehog signaling, which is vital for bone formation. Studies in the human CB-MB pair provided direct evidence that maternal high cholesterol levels are inversely associated with bone formation in newborn offspring. Together, our study confirms that maternal HC diet causes nonreversible derogatory changes in offspring’s skeleton and downregulates hedgehog signaling components in their bones.

## Experimental procedures

### Reagents and chemicals

Cell culture media and necessary supplements, cholesterol, Alizarin S-red, calcein, leucocyte alkaline staining kit, and bromodeoxyuridine (BrdU) cell proliferation kit were obtained from Sigma and Invitrogen. Antibodies were procured from CST, Abcam, and Santa Cruz technology. Cholesterol, Triacylglycerol, HDL-cholesterol, and LDL-cholesterol estimation kits were obtained from Abcam. Osteocalcin estimation kit was obtained from Ray Biotech. P1NP (Pro-collagen Type 1 N terminal propeptide) and CTX (C-Terminal type-1 collagen cross-links) were procured from Elab sciences. Other fine chemicals such as p-nitrophenyl phosphate and MTT were procured from SRL.

### Animals and diets

Adult C57BL6/J mice and Swiss Albino mice were inducted into our study after obtaining prior approval from the Institutional animal ethical committee (IAEC No: CFT/IAEC/29/2015). Plugged mice were divided into C and HC groups. Groups were fed either isocaloric AIN-93G diet as C or HC diet (0.5% cholesterol) throughout their pregnancy and lactation (Table S1). The offspring were weaned on a control diet and maintained till their sacrifice. Intraperitoneal calcein injections were given (20 mg/kg body weight) on day 6 and day 2 before sacrifice. The mice were fasted overnight before the sacrifice. Blood was collected in vacutainers; serums were separated and stored at −80 °C. Long bones, vertebras, and other tissues were also collected for further analysis.

### Skeletal preparation and analysis

Skeletal preparation was performed using a previously published method (77, 78). Briefly, E18.5 embryos were submerged in 60 °C water for 30 to 60 s, followed by removal of skin, epidermis, and viscera. The fetus was then immediately fixed in 100% ethanol overnight. Later, the specimen was stained with 0.2% alcian blue at room temperature for 8 to 10 h followed by washes with 70% and 95% ethanol to remove excess colouration. To preclear the extra tissues, the specimen was incubated in 1% KOH and transferred to 0.5% alizarin red solution for 4 to 5 h. This leads to transparency and better visualization of skeletal elements. Next, the specimen in a step-wise manner was transferred to 100% glycerol for final storage. Images were taken, and ImageJ software was used for analysis. We assessed at least four litters with a minimum of three animals per litter.

### μ-CT measurement of bone microarchitectural parameters

Sky scan 1076 μ-CT scanner was used to scan excised bone samples following the previously published protocols (77, 79). Briefly, excised bones were scanned at an X-ray potential of 45 kV and a current of 220 μA. A rotation step of 0.5 and pixel size of 9 μm was used to acquire the projections. Sky Scan Recon software was used to reconstruct the images with smoothing of 1, a ring artifact reduction of 6, a beam-hardening correction of 35%, and a defect pixel masking of 50%. The samples were then reoriented using Data Viewer to measure the same region for each sample. Regions of interest were defined for trabecular and cortical bone using CTAn Analyzer. Trabecular bone measurement of secondary spongiosa was done in a region with length 15% of total bone length, starting 10% proximal to the growth plate (distal metaphysis). For cortical bone, a 15% long region was measured at a distance 30% proximal to the growth plate. The threshold for trabecular bone was 0.219 mg/cm^3^ calcium hydroxyapatite, and for cortical bone, it was 0.632 mg/cm^3^ calcium hydroxyapatite. Trabecular and cortical BMD of both femur and tibia was evaluated using hydroxyapatite phantom rods of 4 mm in diameter with known BMD as reference. 3D models of both femur and tibia were drawn using CTvol software.

### MCOBs culture

Following previously published protocols, the mouse primary calvarial osteoblast cell culture was carried out (80). Briefly, 6 to 8 calvaria were harvested from newborn pups. Sutures were carefully removed, and the calvarium was scraped gently to remove adherent tissues. Pooled calvaria was digested five times in a row for 15 min each in medium containing 0.1% collagenase and 0.1% dispase solution. The supernatant from the first digestion was discarded, and further fractions from all four digestions were collected and cultured in complete osteoblast growth medium comprising α-minimum essential medium, 10% fetal bovine serum, 1% antibiotic antimycotic solution, 1 mM sodium pyruvate, and 1% nonessential amino acids until they reached 90% confluence for further experiments.

### BrdU cell proliferation assay

BrdU assay was performed following a previously published protocol (81). Briefly, isolated MCOBs from C and HC groups were seeded in 96-well plates (4 × 10^3^ cells/well) and allowed to grow for 24 h in growth medium. After 24 h, cells were labeled with BrdU and fixed, and BrdU assay was performed using the manufacturer’s protocol (Sigma).

### Alkaline phosphatase differentiation assay

Alkaline phosphatase (ALP) assay and staining were performed according to a previously published protocol (82, 83). Briefly, MCOBs from C and HC groups were seeded in 96-well plates (4 × 10^3^ cells/well) and cultured for 48 h in osteoblast differentiation medium containing α-minimum essential medium supplemented with 10% fetal bovine serum, 1 mM sodium pyruvate, 10 mM β glycerophosphate and 50 mg/ml ascorbic acid, 1% penicillin or streptomycin, and 1% NEA. After incubation, cells were freeze fractured, p-nitrophenyl phosphate was added as substrate, and the absorbance was measured at 405 nm. The absorption was normalized with the protein content of the cells and represented as ALP activity per mg of protein.

For ALP staining, MCOBs were seeded in 12-well plates (25 × 10^3^ cells/well) maintained for 8 to 10 days in osteoblast differentiation medium. The ALP staining kit (Sigma) was used to stain the fixed cells according to the manufacturer's protocol.

### Mineralization

Mineralization assay was performed as per the previously established protocol (81). MCOBs were seeded in 12-well plates (50 × 10^3^ cells/well) in osteoblast differentiation medium and maintained for 21 days. At the end, cells were fixed and stained with alizarin red. Cetyl pyridinium chloride, 10%, extraction was performed, and absorbance was measured at 570 nm.

### *Ex vivo* osteoblast and osteoclast culture from bone marrow cells

Both the primary osteoblast and osteoclast cultures from BMCs were performed according to the previously published protocols (77, 84). BMCs from the femur and tibia were isolated and cultured. BMCs were seeded in 12-well plates for osteoblast differentiation and maintained for 21 days in osteoblast differentiation medium supplemented with 10^−7^ M dexamethasone.

For osteoclast culture, cells were allowed to differentiate in the presence of receptor activator of nuclear factor κB ligand (RANKL-50 ng/ml) and monocyte/macrophage colony-stimulating factor (m-CSF-30 ng/ml) in 96-well plates for TRAP staining and 12-well plates for RNA isolation.

### Histomorphometric analysis of vertebrae

Excised undecalcified vertebral samples were embedded in methyl methacrylate and sectioned on Leica microtome (77). Von Kossa staining was performed to estimate the percent bone volume (BV/TV) in LV4 trabecular region, excluding primary spongiosa. Calcein double labeling was performed to determine the mineral apposition rate and bone formation rate/bone surface (BFR/BS) in the trabecular region of LV4 using fluorescence microscopy. Toluidine blue staining was used to calculate osteoblast number per trabecular bone area (N.Ob/Tb.B.Ar), osteoblast number per bone perimeter (N.Ob/B.Pm), and osteocyte number per trabecular bone area (N. Os/Tb. B.Ar) in the trabecular region of the LV4. TRAP staining was performed to count the osteoclast surface per bone surface (Oc.S/BS) and osteoclast cell number per trabecular bone area (Oc.N/Tb.B.Ar) in the trabecular region of the LV4. Histomorphometric analyses were performed by using the Osteomeasure analysis system (Osteometrics).

### qPCR analysis

Total RNA was isolated from MCOBs and long bones using trizol reagent following previously described protocols (82, 85). For RNA isolation from bones, tibias were cleaned to remove muscles and bone marrow was flushed, later homogenized in Trizol, followed by RNA extraction following the manufacturer's protocol. An equal amount of RNA was converted into cDNA using high-capacity cDNA synthesis kit. SYBR green chemistry was used for quantitative determination and the relative expression of all genes using specific primers (Table S2). β–Actin was used as the loading control.

### Western blotting

Western blot analysis was performed according to previously published protocols (79). Protein was harvested from either MCOBs or long bones by using protein lysis buffer. Protein was quantified, and an equal amount of protein was separated on 10% SDS PAGE, transferred to PVDF membranes, and developed with specific antibodies. Later, the expression of genes was normalized with β-actin.

### Human cross-sectional study

Mysore Medical College and Research Institute (MMC&RI) reviewed and approved our study protocol (IHEC No: MMC EC 25/11/2016). Samples were collected from the subjects admitted in the obstetrics and gynecology ward of MMC&RI. Informed consent was taken from respective subjects before sampling. Both maternal blood (MB) and cord blood (CB) dyad samples were collected at delivery time into vacutainers. Serum was separated carefully and stored at −80 °C for further experiments. A total of 200 samples were collected, and 65 were found suitable for the study after qualifying our exclusion and inclusion criteria (Fig. S1). Exclusion criteria: preterm deliveries, gestational diabetes, hypertension, pre-eclampsia, and other pathological conditions. Inclusion criteria: female term pregnancies from normal, borderline, and hypercholesteraemic mothers. Human blood parameters were performed using standard kits following the manufacturer's protocol (shown in later sections. Based on maternal cholesterol levels, samples were classified into three groups, i.e., normal cholesterol (160–200 mg/dl), borderline/level cholesterol (200–240 mg/dl), and high cholesterol >240 mg/dl. Next, we analyzed bone turnover markers from the serum of cord blood samples.

### Analysis of bone turnover markers

Mouse and human P1NP (Pro-collagen Type 1 N terminal propeptide) and CTX (C-Terminal type-1 collagen cross-links) were assessed by using ELISA kits, following the manufacturer's protocol (Elab sciences).

### Serum cholesterol measurements

Total cholesterol, HDL, and LDL content were determined from serum samples using the HDL and LDL Cholesterol enzymatic Assay Kit (Agappe Diagnostics Ltd), following the manufacturer's protocols.

### Statistical analysis

All the results were represented as mean ± SEM. Statistical analysis was performed by using GraphPad Prism eight software. All animal experiments were performed with n ≥6 mice/group. Cell culture studies were repeated thrice independently with a minimum of three replicates. Paired Student’s *t* test was performed between two experimental groups, and one-way ANOVA was performed between more than two experimental groups, followed by Tukey's post hoc test. Human study data were tested for normality and equal variance by the Shapiro–Wilk test. Correlations between MB and CB were determined using the Pearson correlation coefficient. A value of ≤0.05 was considered to be statistically significant. ANOVA was used to compare normal, borderline, and hypercholesterolemic subjects. A *p* <0.05 was considered statistically significant.

## Data availability

All data described are located in this article.

## Supporting information

This article contains supporting information.

## Conflict of interest

The authors declare that they have no conflicts of interest with the contents of this article.
