# Supplementary material for: Maternal high-cholesterol diet negatively programs offspring bone development and downregulates hedgehog signaling in osteoblasts
Source: J Biol Chem. 2022 Aug 2;298(9):102324. doi: 10.1016/j.jbc.2022.102324 (PMC9440389; doi:10.1016/j.jbc.2022.102324)
Supplement: Supporting information [file mmc1.docx]

**Supplementary Table 1. Diet composition chart**

|  | **Control** | **HC** |
| --- | --- | --- |
| **Constituents** | **Per Kg** | **Per Kg** |
| **Starch** | 450 g | 450 g |
| **Cellulose** | 50 g | 50 g |
| **Sucrose** | 144 g | 144 g |
| **Casein (85 %)** | 236 g | 230 g |
| **Soya oil** | 70 g | 70 g |
| **Mineral mix** | 35 g | 35 g |
| **Vitamin mix** | 10 g | 10 g |
| **Methionine** | 03 g | 03 g |
| **Choline chloride** | 02 g | 02 g |
| **Cholesterol** | - | 05 g |
| **t- butyl 1- hydroquinone** | 0.014 g | 0.014 g |
| **Bile salts**  **(Cholic acid 45%)** | - | 1.25 g |

**Supplementary Table 2. List of primer sequence used for q RT PCR**

| Gene name | Primer sequence 5’ – 3’ |
| --- | --- |
| *β- actin* | F- ATGACCCAACCGAGAAGG |
|  | R-CGGCCAAGTCTTAGAGTTGTTG |
| *Runx2* | F- GACTGTGGTTACCGTCATGGC |
|  | R-ACTTG GTTTTTCATAACAGCGGA |
| *Alp* | F- CAGCGGGTAGGAAGCAGTTTC |
|  | R- CCCTGCACCTCATCCCTGA |
| *Col1a1* | F- GCTCCTCTTAGGGGCCACT |
|  | R- CCACGTCTCACCATTGGGG |
| *ATF4* | F- CCTGAACAGCGAAGTGTTGG |
|  | R- TGGAGAACCCATGAGGTTTCAA |
| *Bmp2* | F- GGCCGAAGGTGGATTCTCC |
|  | R- GTCGGGTGTGTTATTGACATACA |
| *Osx* | F- ATGGCGTCCTCTCTGCTTG |
|  | R- TGAAAGGTCAGCGTATGGCTT |
| *Trap* | F- CTTGTGGACGAAAATATGTGGCT |
|  | R- GACTTTCCTGTCGAATGCACT |
| *Ctsk* | F- GAAGAAGACTCACCAGAAGCAG |
|  | R- TCCAGGTTATGGGCAGAGATT |
| *C- fos* | F-TTGAGCGATCATCCCGGTC |
|  | R- GCGTGAGTCCATACTGGCAAG |
| *Opg* | F-ACAAGTGGCTGTGCTGTGC |
|  | R- CGGTTTCTGGGTCATAATGC |
| *Rankl* | F- GTACTTTCGAGCGCAGATGG |
|  | R- CAGAGTCGAGTCCTGCAAACC |

**Supplementary Table 3. Serum and liver lipid profile in the C and HC dams**

| **Serum cholesterol levels** | | | | |
| --- | --- | --- | --- | --- |
| **C57bl6/J mice** | | | | |
| **Parameters** | **At delivery** | | **During weaning** | |
|  | **C** | **HC** | **C** | **HC** |
| **Cholesterol (mg/dl)** | 180.04 ± 2.39 | 277.01 ± 9.64 *** | 160.39 ± 4.97 | 215.90 ± 19.07 * |
| **Triglycerides (mg/dl)** | 73.56 ± 3.57 | 103.20 ± 6.87* | 73.72 ± 3.80 | 98.95 ± 2.80 *** |
| **LDL-Ch (mg/dl)** | 16.42 ± 1.96 | 83.79 ± 4.66 **** | 13.04 ± 1.94 | 51.30 ± 9.07** |
| **HDL-Ch (mg/dl)** | 148.33 ± 3.74 | 172.32 ± 10.58* | 132.6 ± 5.11 | 144.81± 12.09 |
| **Swiss Albino mice** | | | | |
| **Cholesterol (mg/dl)** | 243.1 ± 25.6 | 520.6 ± 34.03 *** | 221.07 ± 8.0 | 448.05 ± 25.38 **** |
| **Triglycerides (mg/dl)** | 61.12 ± 4.54 | 83.24 ± 7.11* | 69.02 ± 5.22 | 99.55 ± 2.76 ** |
| **LDL-Ch (mg/dl)** | 30.40 ± 6.76 | 215.88 ± 25.45**** | 28.90 ± 3.70 | 205.07 ± 24.67**** |
| **HDL-Ch (mg/dl)** | 200.93 ± 30.0 | 290.03 ± 25.6* | 179.1 ± 8.01 | 220.07 ± 6.65** |
| **Liver cholesterol levels** | | | | |
| **C57bl6/J mice** | | | | |
| **Cholesterol (mg/dl)** | 209.07 ± 5.82 | 443.7 ± 13.22 *** | 195.85 ± 7.6 | 418.2 ± 11.2 *** |
| **Triglycerides (mg/dl)** | 79.56 ± 3.57 | 103.20 ± 6.87* | 75.49 ± 3.45 | 99.88 ± 2.67*** |
| **LDL-Ch (mg/dl)** | 74.82 ± 2.92 | 240.78 ± 16.69**** | 66.38 ± 2.71 | 215.5 ± 6.33**** |
| **HDL-Ch (mg/dl)** | 118.33 ± 3.74 | 182.32 ± 10.58*** | 114.36 ± 5.50 | 182.37 ± 6.37** |
| **Swiss Albino mice** | | | | |
| **Cholesterol (mg/dl)** | 272.78 ± 9.64 | 539.09 ± 16.84 **** | 217.04 ± 4.82 | 416.64 ± 6.74 *** |
| **Triglycerides (mg/dl)** | 78.25 ± 2.31 | 107.77 ± 3.59*** | 53.28 ± 7.86 | 109.38 ± 15.95*** |
| **LDL-Ch (mg/dl)** | 62.62 ± 5.95 | 258.38 ± 10.90*** | 34.95 ± 5.10 | 202.835 ± 29.23*** |
| **HDL-Ch (mg/dl)** | 194.5 ± 16.68 | 250.5 ±17.90* | 171.43 ± 24.69 | 191.93 ± 27.73** |

Each parameter represents pooled data from 6 mice/group. Values are expressed as mean±S.E.M

(^*^ P < 0.05, ^**^ P < 0.01, ^***^ P < 0.001).

**Supplementary Table 4. Physiological and 2D µ CT data of 12 weeks offspring’s (C57BL6/J mice)**

| ***Parameters*** | **Female** | | **Male** | |
| --- | --- | --- | --- | --- |
|  | **C** | **HC** | **C** | **HC** |
| ***Body length(mm)*** | 85.90 ± 0.55 | 82.10 ± 0.96* | 86.26 ± 0.72 | 83.01 ± 0.61** |
| ***Body weight (g)*** | 28.89 ± 0.91 | 31.56 ± 1.05* | 29.56 ± 1.02 | 33.54 ± 1.24* |
| ***2d µCT measurements of femur cortical*** | | | | |
| ***BMD (mg/cm^3^)*** | 2.53 ± 0.007 | 1.95 ± 0.24 * | 2.52 ± 0.05 | 1.75 ± 0.32 * |
| ***T. Ar (mm^2^)*** | 2.55 ± 0.05 | 2.28 ± 0.27 | 2.58 ± 0.07 | 1.93 ± 0.35 * |
| ***T. Pm (mm)*** | 6.35 ± 0.07 | 5.39 ± 0.42* | 7.22 ± 0.835 | 5.96 ± 0.21 * |
| ***B. Ar (mm^2^)*** | 1.04 ± 0.022 | 0.76 ± 0.086** | 0.092 ± 0.012 | 0.83 ± 0.027* |
| ***B. Pm (mm)*** | 1.05 ± 0.02 | 0.80 ± 0.05*** | 1.10 ± 0.10 | 0.86 ± 0.03 * |
| ***Cs. Th (mm)*** | 0.17 ± 0.012 | 0.13 ± 0.010* | 0.15 ± 0.0007 | 0.14 ± 0.003* |
| ***Po (cl) %*** | 44.89 ± 4.03 | 54.43 ± 5.008* | 63.79 ± 0.44 | 69.11 ± 5.30 |
| ***MMI (mm^4^)*** | 0.70 ± 0.05 | 0.58 ± 0.05 | 0.80 ± 0.06 | 0.61 ± 0.04 * |
| ***2d µ CT measurements of tibia cortical*** | | | | |
| ***BMD (mg/cm^3^)*** | 1.28 ± 0.20 | 1.010± 0.017*** | 1.84 ± 0.029 | 1.46 ± 0.03 * |
| ***T. Ar (mm^2^)*** | 1.74 ± 0.010 | 1.12 ± 0.18* | 1.90 ± 0.02 | 1.75 ± 0.010 |
| ***T. Pm (mm)*** | 7.14 ± 0.23 | 5.95 ± 0.27** | 7.40 ± 0.0107 | 6.62 ± 0.30* |
| ***B. Ar (mm^2^)*** | 0.085 ± 0.07 | 0.64 ± 0.056* | 0.88 ± 0.02 | 0.64 ± 0.08 * |
| ***B. Pm (mm)*** | 11.41 ± 0.13 | 11.09 ± 0.26 | 12.27 ± 0.14 | 11.16 ± 0.46* |
| ***Cs. Th (mm)*** | 0.17 ± 0.002 | 0.11 ± 0.018 * | 0.16 ± 0.006 | 0.11 ± 0.009*** |
| ***Po (cl) %*** | 48.50 ± 0.83 | 65.33 ± 1.3*** | 54.63 ± 1.28 | 60.33 ± 1.10 * |
| ***MMI (mm^4^)*** | 0.48 ± 0.04 | 0.38 ± 0.03 | 0.52 ± 0.01 | 0.47 ± 0.049 |

Each parameter represents pooled data from 6 mice/group. Values are expressed as mean±S.E.M

(^*^ P < 0.05, ^**^ P < 0.01, ^***^ P < 0.001).

BMD - Bone mineral density, T.Ar – Periosteal area, T.Pm- Periosteal perimeter, B.Ar- Cortical mean cross-sectional area, B.Pm – Cortical bone perimeter, Cs.Th – Cortical thickness, Po – Cortical porosity, MMI – Mean polar moment of inertia

**Supplementary Table 5. Physiological and 3d µ CT parameters of trabecular bone of 6, 12 and 24-week C and HC offspring of both female and male**

| ***Parameters*** | **6 W** | | | | **12 W** | | | | **24 W** | | | |
| --- | --- | --- | --- | --- | --- | --- | --- | --- | --- | --- | --- | --- |
|  | **F** | | **M** | | **F** | | **M** | | **F** | | **M** | |
|  | **C** | **HC** | **C** | **HC** | **C** | **HC** | **C** | **HC** | **C** | **HC** | **C** | **HC** |
| ***Body length (mm)*** | 92.62±0.68 | 90.22±0.41* | 94.22±0.3 | 89.54±0.5*** | 97.13±0.26 | 94.32±0.86* | 99.09±0.37 | 95.45±0.46*** | 101.96±0.71 | 99.46±0.63* | 104.74±0.39 | 101.14±0.66*** |
| ***Body weight (g)*** | 28.90±1.25 | 32.0±0.39 * | 31.75±0.73 | 35.37±0.47** | 30.15±1.02 | 35.47±1.01** | 34.32±0.77 | 41.70±1.21** | 36..0±0.52 | 40.66±1.29** | 51.46±1.3 | 53.99±0.726* |
| ***3D micro CT measurements of distal femur metaphysis*** | | | | | | | | | | | | |
| ***Tb. Th (µm)*** | 0.08±0.005 | 0.05±0.006** | 0.06±0.002 | 0.058±0.0015** | 0.1001±0.003 | 0.06±0.0004** | 0.08±0.005 | 0.05±0.0004*** | 0.701±0.002 | 0.065±0.0014 | 0.070±0.004 | 0.058±0.0007* |
| ***Tb. Sp (µm)*** | 0.187±0.01 | 0.22±0.0069* | 0.25±0.008 | 0.344±0.016*** | 0.198±0.002 | 0.234±0.008** | 0.20±0.037 | 0.23±0.037* | 0.23±0.0081 | 0.279±0.010 | 0.25±0.006 | 0.27±0.010* |
| ***Tb. N (1/µm)*** | 3.58±0.24 | 2.58±0.11** | 2.71±0.112 | 1.70±0.116*** | 3.20±0.07 | 2.41±0.196** | 2.88±0.23 | 2.018±0.14** | 2.77±0.20 | 1.979±0.202* | 2.09±0.14 | 1.47±0.145* |
| ***Tb.pf*** | 6.211±2.24 | 17.7±0.87*** | 13.45±0.84 | 21.79±1.45*** | 7.37±0.35 | 16.65±1.27 *** | 15.6±1.16 | 22.15±0.75*** | 10.97±1.49 | 17.59±1.60* | 16.49±1.61 | 24.55±1.83** |
| ***SMI*** | 1.37±0.17 | 2.04±0.04** | 1.89±0.07 | 2.29±0.051*** | 1.70±0.05 | 2.10±0.09** | 2.017±0.015 | 2.28±0.028 *** | 1.92±0.157 | 2.15±0.112 | 2.09±0.077 | 2.41±0.079* |
| ***Con Dn(mm^-3^)*** | 204.13±11.7 | 105.29±8.6** | 155.4±4.3 | 68.99±7.57 *** | 165.78±7.15 | 97.09±8.16 *** | 157.58±8.7 | 71.94±8.7* | 89.40±9.35 | 61.54±5.03* | 73.88±10.48 | 44.34±6.47* |
| ***MMI*** | 0.128±0.031 | 0.052±0.007* | 0.058±0.015 | 0.038±0.005 | 0.0733±0.011 | 0.03±0.005*** | 0.16±0.03 | 0.049±0.007*** | 0.04±0.008 | 0.022±0.001* | 0.972±0.019 | 0.043±0.004* |
| ***3D micro CT measurements of Proximal tibia*** | | | | | | | | | | | | |
| ***Tb. Th (µm)*** | 0.062±0.008 | 0.05±0.001** | 0.05±0.001 | 0.048±0.002** | 0.006±0.0095 | 0.05±0.008** | 0.63±0.010 | 0.055±0.0078 | 0.066±0.009 | 0.05±0.008** | 0.06±0.10 | 0.055±0.007 |
| ***Tb. Sp (µm)*** | 0.29±0.16 | 0.30±0.015 | 0.34±0.013 | 0.471±0.04* | 0.279±0.041 | 0.31±0.045* | 0.270±0.040 | 0.32±0.04* | 0.279±0.041 | 0.31±0.045* | 0.270±0.040 | 0.322±0.049* |
| ***Tb. N (1/µm)*** | 1.92±0.23 | 1.41±0.150 | 1.14±0.04 | 0.65±0.129** | 1.71±0.268 | 1.07±0.169** | 1.72±0.29 | 1.01±0.20* | 1.71±0.268 | 1.07±0.169** | 1.72±0.29 | 1.018±0.204* |
| ***Tb.pf*** | 21.99±2.38 | 28.30±1.68* | 27.79±0.891 | 36.7±3.21* | 24.40±3.678 | 31.385±4.34** | 23.20±3.37 | 32.49±4.86*** | 24.40±3.67 | 31.38±4.43** | 23.20±3.37 | 32.49±4.86*** |
| ***SMI*** | 2.29±0.07 | 2.48±0.034* | 2.53±0.010 | 2.785±0.041*** | 2.39±0.35 | 2.72±0.39** | 2.39±0.34 | 2.78±0.40*** | 2.39±0.35 | 2.72±039** | 2.39±0.34 | 2.78±0.40*** |
| ***Con Dn(mm^-3^)*** | 123.3±19.2 | 32.86±6.19** | 33.078±3.24 | 18.8±2.10** | 79.74±13.135 | 34.71±5.60*** | 62.61±9.93 | 30.80±5.244*** | 79.74±13.13 | 34.71±5.60*** | 62.61±9.93 | 30.80±5.244*** |
| ***MMI*** | 0.62±0.13 | 0.23±0.04** | 0.37±0.058 | 0.18±0.030*** | 0.63±0.082 | 0.25±0.009*** | 0.47±0.06 | 0.17±0.016** | 0.07±0.011 | 0.03±0.005*** | 0.016±0.03 | 0.04±0.007*** |

Each parameter represents pooled data from 6 mice/group, and values are expressed as mean±SEM. *P<0.05, **P<0.01, ***P<0.001.

Tb.Th - trabecular thickness, Tb.Sp - trabecular separation, Tb.N - trabecular number, Tbpf - trabecular pattern factor, SMI - structure model index, Conn.Dn - connection density, MMI - Mean polar moment of inertia

**Supplementary Table 6. 2d µ CT parameters of cortical bone of 6,12 and 24-week C and HC offspring of both female and male.**

| ***Parameters*** | 6 W | | | | 12 W | | | | 24 W | | | |
| --- | --- | --- | --- | --- | --- | --- | --- | --- | --- | --- | --- | --- |
|  | F | | M | | F | | M | | F | | M | |
|  | C | HC | C | HC | C | HC | C | HC | C | HC | C | HC |
| ***2D micro CT measurements of femur cortical*** | | | | | | | | | | | | |
| ***BMD (mg/cm^3^)*** | 1.17±0.03 | 1.05±0.07** | 1.122±0.01 | 1.06±0.007* | 1.30±0.02 | 1.2±0.014** | 1.37±0.21 | 1.401±0.17*** | 1.277±0.019 | 1.273±0.005 | 1.280±0.02 | 1.21±0.24* |
| ***T. Ar (mm^2^)*** | 2.25±0.039 | 1.99±0.04*** | 2.166±0.033 | 1.9±0.05* | 1.89±0.100 | 1.15±0.023$ | 1.99±0.11 | 1.45±0.14* | 2.07±0.04 | 1.93±0.043* | 1.94±0.06 | 1.85±0.04 |
| ***T. Pm (mm)*** | 5.79±0.064 | 5.37±0.06** | 5.88±0.101 | 5.4±0.07** | 5.75±0.83 | 5.33±0.05*** | 5.75±0.83 | 5.33±0.055*** | 5.60±0.06 | 5.39±0.04* | 5.40±0.08 | 5.26±0.05 |
| ***B. Ar (mm^2^)*** | 0.97±0.059 | 0.77±0.012** | 0.909±0.04 | 0.66±0.015*** | 1.18±0.03 | 0.9±0.022$ | 1.10±0.04 | 1.05±0.055$ | 1.13±0.029 | 1.08±0.03 | 1.059±0.070 | 0.89±0.021* |
| ***B. Pm (mm)*** | 10.28±0.224 | 9.83±0.221 | 10.31±0.083 | 8.66±0.73* | 11.10±1.97 | 9.67±0.100** | 10.53±1.78 | 9.64±0.126** | 10.67±0.20 | 9.38±0.25** | 9.61±0.23 | 9.22±0.094 |
| ***Cs. Th (mm)*** | 0.19±0.012 | 0.15±0.002** | 0.17±0.02 | 0.135±0.019* | 0.22±0.009 | 0.17±0.0026*** | 0.23±0.008 | 0.156±0.0001$ | 0.211±0.003 | 0.20±0.0031 | 0.22±0.01*** | 0.18±0.0018* |
| ***Po (Cl)%*** | 53.64±1.88 | 57.05±1.8 | 59.81±2.733 | 61.92±1.69 | 46.81±1.39 | 53.61±0.40* | 44.78±1.46 | 60.03±0.35*** | 45.17±1.5 | 47.42±1.33 | 46.93±2.69 | 52.41±0.77* |
| ***MMI (mm^4^)*** | 0.55±0.34 | 0.42±0.021* | 0.55±0.079 | 0.30±0.049$ | 0.49±0.086 | 0.46±0.018 | 0.575±0.11 | 0.39±0.011* | 0.58±0.015 | 0.49±0.020** | 0.55±0.035 | 0.40±0.017** |
| ***2D micro CT measurements of Tibia Cortical*** | | | | | | | | | | | | |
| ***BMD (mg/cm^3^)*** | 1.07±0.02 | 1.02±0.014* | 1.08±0.007 | 1.034±0.011** | 1.30±0.08 | 1.16±0.012 | 1.20±0.02 | 1.06±0.013** | 1.21±0.010 | 1.20±0.006 | 0.169±0.013 | 0.122±0.007* |
| ***T. Ar (mm^2^)*** | 1.59±0.06 | 1.47±0.018 | 1.80±0.04 | 1.66±0.042* | 1.77±0.671 | 1.465±0.55*** | 1.82±0.68 | 1.55±0.58** | 1.62±0.05 | 1.51±0.072 | 1.82±0.090 | 1.415±0.041** |
| ***T. Pm (mm)*** | 6.1±0.88 | 5.86±0.834* | 6.3±0.109 | 6.19±0.103 | 6.65±2.51 | 6.36±2.40 | 6.82±2.58 | 6.31±2.38** | 6.69±0.108 | 5.68±0.14*** | 6.80±0.411 | 6.10±0.19* |
| ***B. Ar (mm^2^)*** | 0.90±0.05 | 0.75±0.014* | 0.89±0.052 | 0.727±0.039* | 1.088±0.411 | 0.86±0.32** | 1.031±0.38 | 0.78±0.29*** | 1.08±0.035 | 1.05±0.04 | 0.97±0.059 | 0.85±0.025* |
| ***B. Pm (mm)*** | 10.18±0.31 | 9.69±0.060 | 11.16±0.18 | 10.51±0.14 | 11.02±4.16 | 9.73±3.6*** | 11.19±4.23 | 10.03±3.79*** | 10.58±0.21 | 9.71±0.22* | 10.68±0.30 | 9.87±0.18* |
| ***Cs. Th (mm)*** | 0.178±0.013 | 0.14±0.003* | 0.16±0.007 | 0.12±0.004*** | 0.207±0.078 | 0.171±0.064 *** | 0.19±0.073 | 0.19±0.072 | 0.25±0.05 | 0.093±0.004 | 0.24±0.05 | 0.165±0.0031 |
| ***Po (Cl)%*** | 39.78±1.83 | 47.28±2.0* | 43.74±2.8 | 54.03±1.86* | 44.34±16.76 | 32.4±12.27** | 50.53±19.10 | 40.37±15.25** | 33.09±0.60 | 37.85±0.5*** | 41.32±1.7 | 42.08±0.58 |
| ***MMI (mm^4^)*** | 0.48±0.205 | 0.35±0.009** | 0.52±0.04 | 0.37±0.021** | 0.54±0.20 | 0.387±0.14** | 0.58±0.220 | 0.41±0.15*** | 0.47±0.05 | 0.51±0.041 | 0.54±0.03 | 0.40±0.046* |

Each parameter represents pooled data from 6 mice/group, and values are expressed as mean±SEM. *P<0.05, **P<0.01, ***P<0.001.

T.Ar – Periosteal area, T.Pm - Periosteal perimeter, B.Ar - Cortical mean cross-sectional area, B.Pm – Cortical bone perimeter, Cs.Th – Cortical thickness, Po – Cortical porosity, MMI – Mean polar moment of inertia

**Supplementary Table 7. Basic anthropometric measurements and clinical characteristics of human subjects (mothers delivered female offspring’s)**

| **Parameters** | **Normal cholesterol (160-200 mg/dL)** | **Border line**  **(200-240 mg/dL)** | **High cholesterol (>240 mg/dL)** |
| --- | --- | --- | --- |
| ***No of Samples*** | 33 | 20 | 12 |
| ***Age of mothers at the time of delivery*** | 22.72 ± 0.68 | 22.74 ± 0.74 | 21.33 ± 0.55 |
| ***Weight gain during pregnancy (kg)*** | 8.39 ± 0.52 | 9.0 ± 0.66 | 9.6 ± 1.33 |
| ***Blood pressure*** | 123/79 | 119/78 | 120/68 |
| ***Gestational age*** | 39 weeks ± 0.25 | 39 weeks ± 0.25 | 39 weeks ± 0.31 |
| ***Birth weight (kg)*** | 2.98 ± 0.56 | 2.98 ± 0.93 | 2.92 ± 0.86 |
| ***Maternal cholesterol*** | 166.42 ± 4.28 | 220.40 ± 2.46 *** | 312.61 ± 22.01*** |
| ***Maternal HDL*** | 41.14 ± 2.46 | 46.16 ± 2.79 | 62.56 ± 8.07 ** |
| ***Maternal LDL*** | 52.74 ± 3.32 | 75.35 ± 5.17 *** | 115.94 ± 12.17 *** |
| ***Maternal TAG*** | 123.74 ± 9.42 | 135.98 ± 12.94 | 171.78 ± 18.41* |
| ***Maternal VLDL*** | 24.74 ± 1.88 | 27.19 ± 2.58 | 34.35 ± 3.68 * |

**Supplementary figure 1. Schematic representation of experimental plan for human cross-sectional correlation study.**

**Maternal blood samples**

Analysis of cholesterol levels along with TAG, VLDL, HDL & LDL

**Cord blood samples**

Analysis of bone turn over markers

Osteocalcin, P1NP & CTX

No of pairs selected - 65 CB samples of female offspring with their respective maternal blood samples

**Study tenure**

Study was conducted from April 2017 to December 2017. Subjects admitted during this period for delivery in Obstetrics and gynaecology department of Mysore medical college, between the age group 21 – 35 was included in our study.

Recurrent infections, history of familial hypercholesterolemia, CVD, Ischemic heart disease, hypertension, diabetes, premature stroke, thyroid disorders, statin treatment, subjects on treatment of steroidal and anti-steroidal drugs.

Healthy full-term pregnancies who delivered female offspring’s.

**Exclusion criteria**

**Inclusion criteria**

Total number of samples collected 250 pairs of CB – MB dyad

**Objective of study**

To correlate bone turnover markers from cord blood samples with maternal blood cholesterol levels collected at the time of delivery.
